# Antibacterial and immunomodulatory activities of insect defensins-DLP2 and DLP4 against multidrug-resistant *Staphylococcus aureus*

**DOI:** 10.1038/s41598-017-10839-4

**Published:** 2017-09-21

**Authors:** Zhanzhan Li, Ruoyu Mao, Da Teng, Ya Hao, Huixian Chen, Xiumin Wang, Xiao Wang, Na Yang, Jianhua Wang

**Affiliations:** 1 Key Laboratory of Feed Biotechnology, Ministry of Agriculture, Beijing, 100081 People’s Republic of China; 2 Gene Engineering Laboratory, Feed Research Institute, Chinese Academy of Agricultural Sciences, Beijing, 100081 People’s Republic of China

## Abstract

Methicillin-resistant *Staphylococcus aureus* (MRSA), are the most frequent cause of sepsis, which urgently demanding new drugs for treating infection. Two homologous insect CSαβ peptides-DLP2 and DLP4 from *Hermetia illucens* were firstly expressed in *Pichia pastoris*, with the yields of 873.5 and 801.3 mg/l, respectively. DLP2 and DLP4 displayed potent antimicrobial activity against Gram-positive bacteria especially MRSA and had greater potency, faster killing, and a longer postantibiotic effect than vancomycin. A 30-d serial passage of MRSA in the presence of DLP2/DLP4 failed to produce resistant mutants. Macromolecular synthesis showed that DLP2/DLP4 inhibited multi-macromolecular synthesis especially for RNA. Flow cytometry and electron microscopy results showed that the cell cycle was arrested at R-phase; the cytoplasmic membrane and cell wall were broken by DLP2/DLP4; mesosome-like structures were observed in MRSA. At the doses of 3‒7.5 mg/kg DLP2 or DLP4, the survival of mice challenged with MRSA were 80‒100%. DLP2 and DLP4 reduced the bacterial translocation burden over 95% in spleen and kidneys; reduced serum pro-inflammatory cytokines levels; promoted anti-inflammatory cytokines levels; and ameliorated lung and spleen injury. These data suggest that DLP2 and DLP4 may be excellent candidates for novel antimicrobial peptides against staphylococcal infections.

## Introduction

In recent years, there has been a rapid emergence of highly antibiotic-resistant bacterial infections in both nosocomial and community settings, which is a major cause of morbidity and mortality worldwide^1, 2^. Multidrug resistant organisms, especially methicillin-resistant *Staphylococcus aureus* (MRSA), which is the most common organism with higher mortality than that of methicillin-sensitive *S*. *aureus* (MSSA)^3, 4^, are expected to become more frequent causes of death than cancer in the coming decades^5^. Despite the urgent need for new antibiotics that are effective against resistant bacteria, new compound sources, such as the large inventory of antimicrobial host defense peptides from different organisms, which have the advantages of the broad spectrum activity, rapid action and low target-based resistance, can open up new avenues for the development of new anti-infectives^6^.

Although insects make up 90% of the total number of animals on earth, insect-derived AMPs only account for approximately 10% of more than 2,830 antimicrobial peptides (AMPs) listed in the Antimicrobial Peptide Database, thus there are more AMPs with activity just waiting to be discovered^7^. Insect AMPs, which are essential components of insect innate immune system, may have potential applications in the medicine field. Most insect AMPs are small cationic molecules that are active against bacteria, virus and fungi^8^. According to structural similarity or unique sequences, insect AMPs can be categorized into four classes: α-helix (cecropin and moricin), Cys-rich (insect defensin and drosomycin), Pro-rich (apidaecin, drosocin, and lebocin), and Gly-rich peptides (attacin)^7^. Defensins, which are composed of 34‒51 residues, are the most widespread AMPs characterized in insects^8^. At present, more than 70 insect defensins have been identified in various arthropods, including Sarcophaga, Sapecin, and Aedes^9–11^. They commonly contain an N-terminal loop, an α-helix, and an antiparallel β-sheet, forming a “cysteine-stabilized alpha beta (CSαβ)” or “loop-helix-sheet” structure^12^. These insect CSαβ defensins are active mainly against Gram-positive bacteria, including *S*. *aureus*, *Micrococcus luteus*, and *Aerococcus viridians* ^7^.

Recently, a novel insect defensin-like peptide DLP4 is isolated and identified in immunized hemolymph and various tissues of black soldier fly *Hermetia illucens* ^13^. The 40-residue DLP4 peptide shares high sequence similarity (60‒67.5%) to other insect defensins, such as sapecin^14^, defensin A^12^, and lucifensin^15^; it exhibited potent antibacterial activity against Gram-positive bacteria including MRSA with the minimal inhibitory concentrations (MICs) of 0.59‒2.34 μM, but not Gram-negative bacteria such as *Escherichia coli*, *Enterobacter aerogenes*, and *Pseudomonas aeruginosa* ^13^. However, little is known about the clinical pharmacology and antibacterial mechanisms of DLP4 and its analogue DLP2.

In this study, the synthesized *DLP2* and *DLP4* genes were firstly expressed in *Pichia pastoris* X-33. The structure, antimicrobial activity, toxicity, resistance and pharmacodynamics of DLP2 and DLP4 against MRSA were systematically evaluated *in vitro*. A possible mechanism of DLP2 and DLP4 against MRSA were further investigated. Additionally, the antibacterial and inflammatory properties of DLP2 and DLP4 were examined in a mouse peritonitis model of MRSA infection.

## Results

### Structure analysis of DLP2 and DLP4

**Figure 1:**
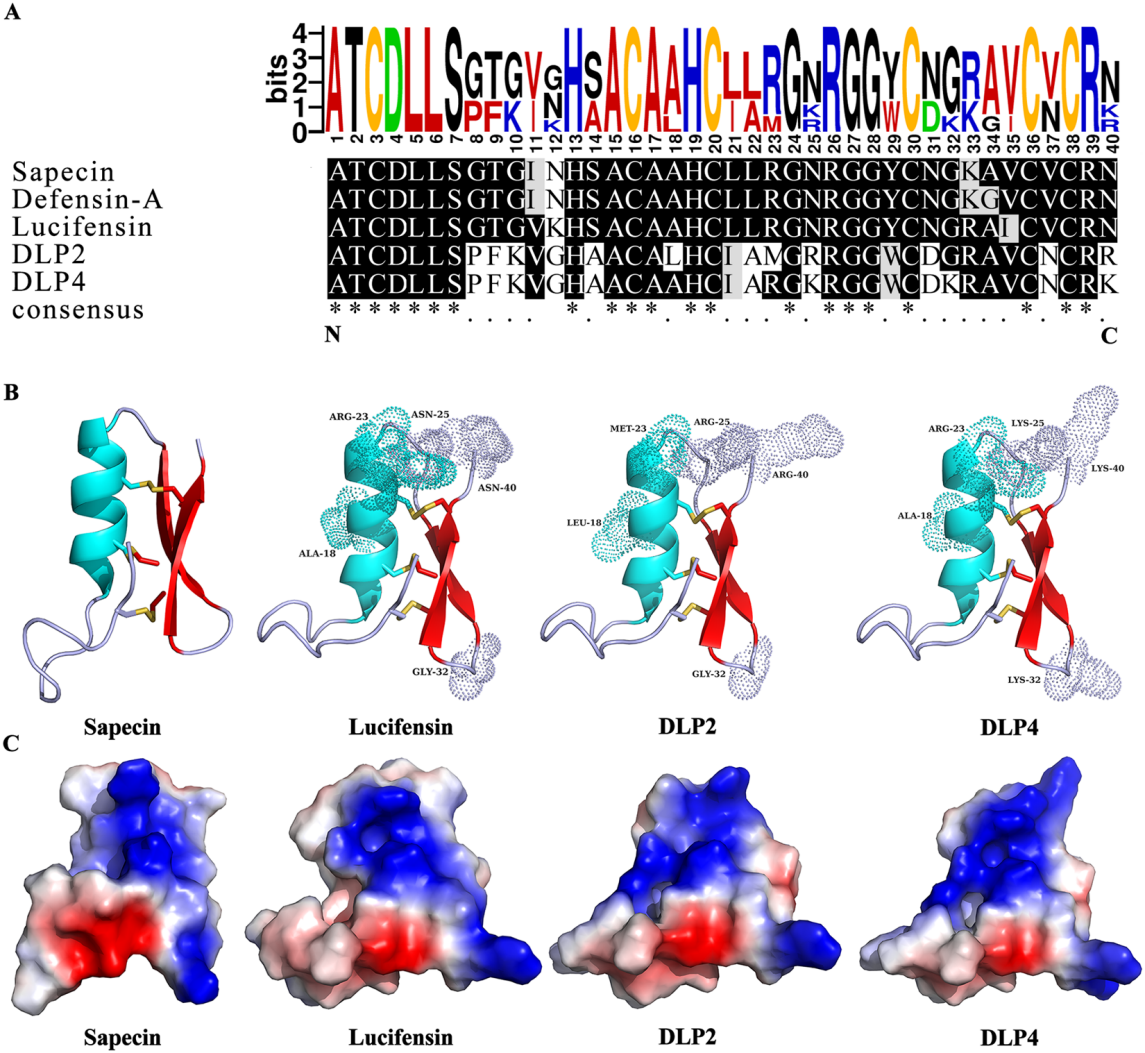
Sequence and structural analysis of DLP2 and DLP4. (**A**) Conservative analysis and sequence alignment of DLP2 and DLP4. The red, black, blue, green, and orange colors represent individual nonpolar amino acids, polar ones, basic ones, acid ones, and cysteine. The “·” under the sequences indicates amino acid differences compared with each other and the “*” indicates the conserved amino acid residues. (**B**) Molecular modeling of DLP2, DLP4, sapecin, and lucifensin. Light dots represent mutation residues. (**C**) Electrostatic surface of DLP2, DLP4, sapecin, and lucifensin. Blue, red and white represent positive, negative, and neutral charge, respectively. Molecular models were generated with PyMOL 1.8.

Conservative analysis and sequence alignment showed that both DLP2 and DLP4 have high sequence similarity (60‒65%) to insect defensins: sapecin^16^, insect defensin A^12^, and lucifensin^8^, which are composed of 40 amino acid residues (Fig. 1A). DLP2 and DLP4 were predicted to adopt the characteristic structure of insect defensins including a loop (residues 1–12), three pairs of disulfide bonds, α-helix (residues 13–23) and an antiparallel β-sheet (residues 28–31 and 34–38) (Fig. 1B), which folding into a typical CSαβ conformation. Additionally, an amphipathic surface can be observed in DLP2, DLP4, sapecin, and lucifensin (Fig. 1C), indicating that these peptides maybe interact with anionic membranes of bacteria.

### Expression, purification, and identification of DLP2 and DLP4

**Figure 2:**
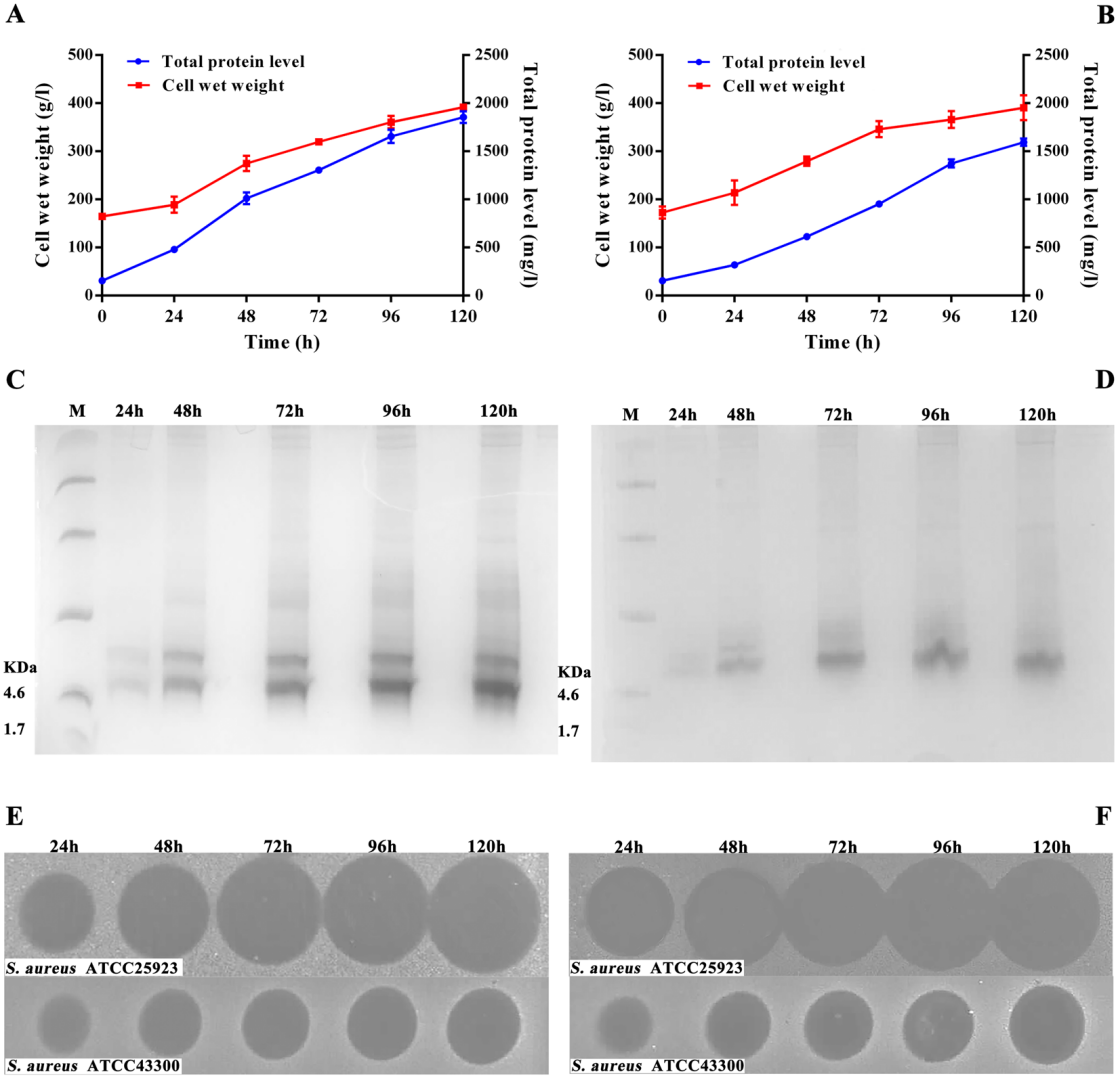
Expression of DLP2 and DLP4 in *P*. *pastoris* X-33 in a 5-l fermentor. (**A**,**B**) Time curve of the concentration of secreted protein levels and cell wet weights during induction. (**A**) DLP2 expression; (**B**) DLP4 expression. Triplicate observations were made, and bars represent the standard error of the mean. (**C**,**D**) Tricine-SDS-PAGE analysis of the fermentation supernatants at different induced time. Lanes M: protein molecular weight marker (5 μl); others lanes: fermentation supernatants (10 μl) of DLP2 (**C**) and DLP4 (**D**) were taken at 24, 48, 72, 96 and 120 h, respectively. The full-length gels are presented in Supplementary Figure 6. (**E**,**F**) The inhibition zones of fermentation supernatant against *S*. *aureus* ATCC25923 and MRSA ATCC43300. Fermentation supernatants of DLP2 (12 μl) (**E**) and DLP4 (20 μl) (**F**) were taken at 0, 24, 48, 72, 96, and 120 h, respectively. The full-length inhibition zones are presented in Supplementary Figure 7.

The codon-optimized linear DLP2 or DLP4 gene digested with *Xho*I and *Xba*I was inserted into pPICZαA and transformed into *E*. *coli* DH5α (Supplementary Fig. S1A and B). The recombinant pPICDLP2 and pPICDLP4 plasmids were linearized by *Bgl*II and transferred into *P*. *pastoris* X-33 competent cells. Transformants with the maximum diameter of inhibition zones against *S*. *aureus* ATCC25923 were selected to be expressed in the shaking flask. The results showed that the expression level and antimicrobial activity of DLP2 and DLP4 were increased with induction time (date not shown), and approximately 175 mg/l of target peptides was obtained from the culture supernatant in the shaking flask after 120 h of induction. To enhance the yield of peptides, high-density cultivation was performed in a 5-l fermentor. As shown in Fig. 2, the total secreted proteins of DLP2 and DLP4 reached 1.856 g/l and 1.6 g/l, and their wet weight of yeast was up to 391.72 g/l and 390.6 g/l, respectively (Fig. 2A and B). The antibacterial activity of DLP2 and DLP4 against *S*. *aureus* ATCC25923 and ATCC43300 (MRSA) displayed time-dependent relationship (Fig. 2E and F).

One-step purification by cation-exchange chromatography with the SP Sepharose FF cation-exchange column was carried out to purify DLP2 and DLP4. The recovery of DLP2 and DLP4 was calculated to be 46.96% and 50.1%, and the corresponding final yields were 873.5 and 801.3 mg/l, respectively. In addition, MALDI-TOF MS analysis indicated that only a target peak of 4,271.78 Da and 4,268.98 Da was obtained from the purified DLP2 and DLP4, respectively (Supplementary Fig. S2), which was consistent with their theoretical molecular values of 4,271 Da and 4,269 Da (Table 1).

**Table 1:** Amino acid sequences and physicochemical properties of DLP2 and DLP4.

Peptides	Sequences^a^	MW (Da)	PI	Charge	Hydrophobicity^b^
DLP2	ATCDLLSPFKVGHAACA**L**HCIA**M**G**R**RGGWCD**G**RAVCNCR**R**	4271.0	8.98	4	0.468
DLP4	ATCDLLSPFKVGHAACA**A**HCIA**R**G**K**RGGWCD**K**RAVCNCR**K**	4269.0	9.38	6	0.354

Note: MW: molecular weight; PI: isoelectric point. ^a^The bold characters indicate different sites of two peptides; ^b^Hydrophobicity is calculated using Heliquest.

### Antimicrobial ability and *in vitro* pharmacodynamics analysis of DLP2 and DLP4

**Figure 3:**
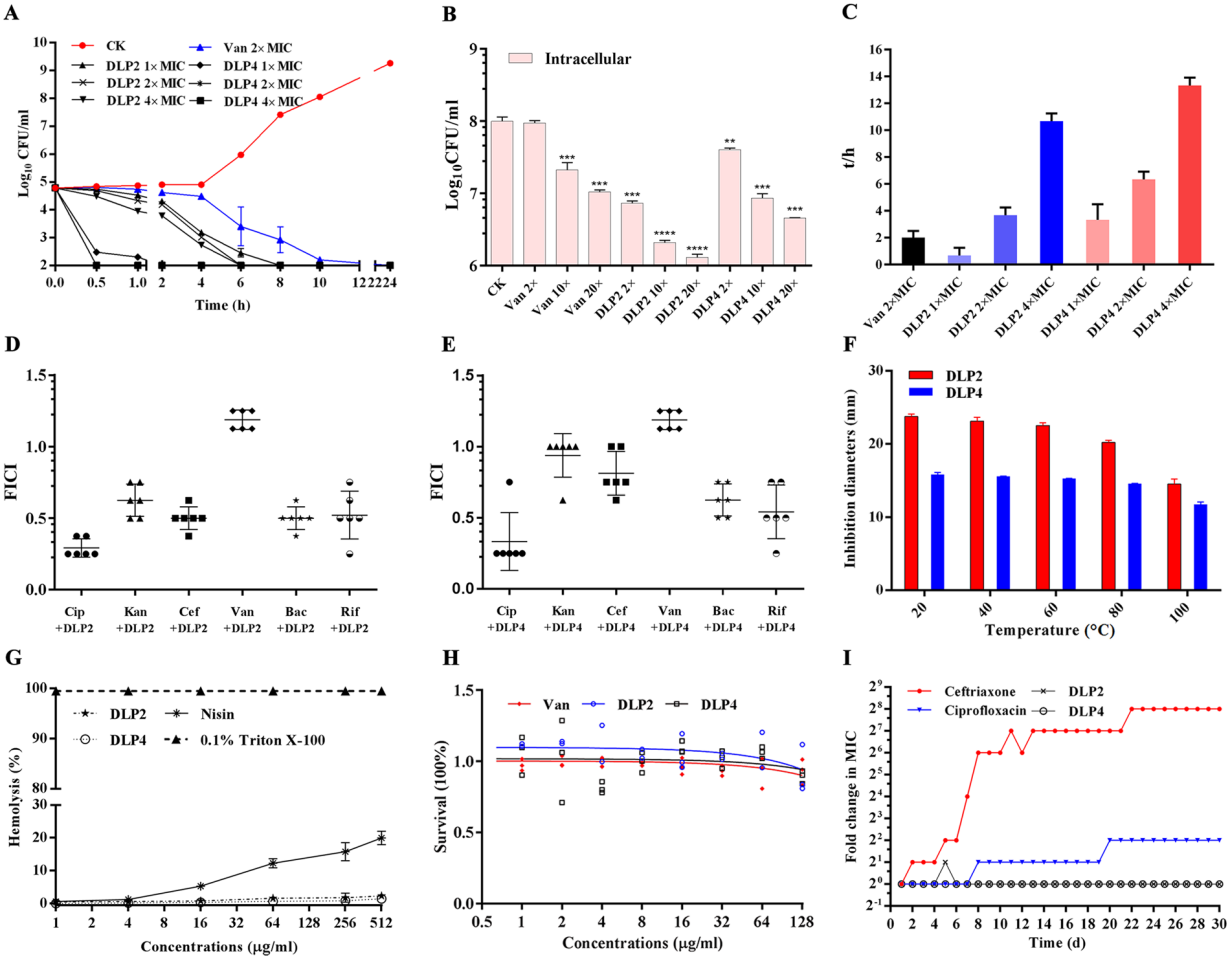
Time-kill curves, stability, toxicity, and pharmacological indexes of DLP2 and DLP4. (**A**) Time-kill curves of the two peptides and antibiotics against MRSA ATCC43300 *in vitro*. CK: without antibiotics and peptides. Results were given as mean ± SD (n = 3). (**B**) Inhibition of intracellular survival of MRSA ATCC43300 in RAW264.7 cells by peptides. PBS and vancomycin were used as negative and positive control, respectively. Results were given as mean ± SD (n = 3). Statistical significance of differences between experimental groups of animals was determined using the one-way ANOVA and Bonferroni multiple comparison. (*) indicates the significance between vancomycin or peptides and CK. **p < 0.01; ***p < 0.001; ****p < 0.0001. (**C**) The PAE (h) of different concentrations of peptides. (**D**,**E**) The FICI of each combination of different antibiotics with DLP2 (**D**) and DLP4 (**E**). Cip, ciprofloxacin; Kan, kanamycin; Cef, ceftriaxone; Van, vancomycin; Bac, bacitracin; Rif, rifampicin. (**F**) Effects of temperature on the peptide activity against *S. aureus* ATCC43300. (**G**) Hemolytic activity of the peptides against fresh mouse red blood cells ﻿(mRBCs). 0.1% Triton X-100 (triangle) served as positive control (100% hemolysis). (**H**) The cytotoxic activity of peptides and antibiotics against RAW264.7 monocytes. The 1 on the *y*-axis represents 100% survival. (**I**) Drug resistance development profiles of *S*. *aureus* exposed to sub-MIC concentrations of DLP2, DLP4, ceftriaxone and ciprofloxacin. A representative of four independent experiments is shown.

As shown in Table 2, DLP2 and DLP4 displayed more potent antibacterial activity against Gram-positive bacteria including MRSA, *Streptococcus suis*, and *Listeria ivanovii* with the MIC values of 0.01‒0.47 μM than plectasin^17^. The MICs of DLP2 were 2- to 4-fold below that of DLP4, indicating the more potent activity of DLP2. However, the two peptides showed very low activity against Gram-negative bacteria such as *E*. *coli* and *Salmonella* with the MICs over 29.97 μM. After the addition of 0.225% to 1.8% NaCl to MHB, the MICs of DLP2, DLP4 and antibiotics against Gram-positive bacteria except for *S*. *suis* CVCC606 did not significantly change (Supplementary Table S1). The time-killing kinetic curve showed that an obvious decrease in bacterial growth appeared after 0.5 h exposure to DLP4, indicating that MRSA ATCC43300 was rapidly killed by DLP4 within 0.5 h (Fig. 3A). Comparably, the antibacterial potency of DLP2 was lower than that of DLP4. The positive control agent vancomycin (2 × MIC) showed the slowest antibacterial rate within 2‒4 h. Moreover, DLP2 and DLP4 could kill intracellular MRSA ATCC43300 in a concentration-dependent manner in RAW264.7 macrophage cells (Fig. 3B). The Log_10_ (CFU/ml) of *S*. *aureus* significantly (p < 0.001) decreased by 0.98, 1.68‒1.89 and 1.06‒1.34, respectively after treatment with 20 × MIC vancomycin, 10 × MIC and 20 × MIC DLP2 or DLP4.

**Table 2:** MIC values of DLP2 and DLP4 against bacteria.

Species and strains	MIC							
	DLP2		DLP4		Vancomycin		Ciprofloxacin	
	μM	μg/ml	μM	μg/ml	μM	μg/ml	μM	μg/ml
Gram-positive bacteria								
*Staphylococcus aureus* ATCC25923^a^	0.01	0.031	0.01	0.062	0.04	0.062	0.08	0.031
*S*. *aureus* ATCC43300^a^	0.12	0.5	0.23	1.0	0.69	1.0	5.44	2.0
*S*. *aureus* ATCC6538^a^	0.12	0.5	0.47	2.0	NT	NT	NT	NT
*S*. *aureus* CICC546^c^	0.23	1.0	0.47	2.0	0.35	0.5	2.72	1.0
*Streptococcus suis* CVCC606^b^	0.93	4.0	1.88	8.0	0.01	0.015	0.045	0.015
*Listeria ivanovii* ATCC19119^a^	0.12	0.5	0.12	0.5	0.35	0.5	1.36	0.5
Gram-negative bacteria								
*Escherichia coli* CVCC1515^b^	>29.97	>128	>29.98	>128	44.17	64	0.04	0.016
*E*. *coli* CICC21530 (serotype O157:H7)^c^	>29.97	>128	>29.98	>128	44.17	64	0.04	0.016
*Salmonella typhimurium* ATCC14028^a^	>29.97	>128	>29.98	>128	>88.34	>128	0.04	0.016
*S*. *enteritidis* CMCC50336^d^	>29.97	>128	>29.98	>128	>88.34	>128	0.04	0.016

Note: NT: not test. ^a^American Type Culture Collection (ATCC); ^b^China Veterinary Culture Collection Center (CVCC); ^c^China Center of Industrial Culture Collection (CICC); ^d^National Center Center for Medical Culture Collection (CMCC). Data were representative of three independent experiments.

The results of postantibiotic effect (PAE) of DLP2 and DLP4 against MRSA ATCC43300 were shown in Fig. 3C. The PAE of DLP2 to *S*. *aureus* was 0.67, 3.67, and 10.67 h, respectively at 1, 2, and 4 × MIC. The PAEs at the same concentrations of DLP4 on *S*. *aureus* were 3.33, 6.33, and 13.33 h, respectively, whereas the PAE of vancomycin at 2 × MIC was 2 h (Fig. 3C), indicating a dose-independent PAE was observed with DLP2 and DLP4 against MRSA ATCC43300.

A synergism was observed in combination of DLP2 and ciprofloxacin, ceftriaxone, and bacitracin against MRSA ATCC43300 with fractional inhibitory concentration index (FICI) ranging from 0.291 to 0.5, and detected between DLP4 and ciprofloxacin (FICI = 0.333) (Fig. 3D and E). However, the FICI values between DLP4 and ceftriaxone and bacitracin were higher than 0.5, indicating additive effects. In addition, kanamycin and rifampicin had the same additive effect with both DLP2 and DLP4, which was different from NZ2114 in combination with kanamycin (with FICI value of 0.3125)^17^. However, the combination interactions of vancomycin with DLP2 and DLP4 demonstrated an indifference effect with FICI values of 1.188 (Fig. 3D and E).

### DLP2 and DLP4 displayed high thermal stability, low toxicity, and no resistance

DLP2 and DLP4 retained a high activity level against *S. aureus* ATCC25923 of greater than 94.9% in a temperature range from 20 °C to 60 °C; 85% and 93% of the activity of DLP2 and DLP4 were remained after a 1-h incubation at 80 °C, and 60‒73.7% of the activity of DLP2 and DLP4 were observed to remain at 100 °C (Fig. 3F), which had higher thermal activity than NZ2114^17^.

The cytotoxicity of DLP2 and DLP4 was determined by the ability to lyse mouse blood erythrocytes and peritoneal macrophages RAW264.7 cells, respectively. The results showed that DLP2 and DLP4 had no significant hemolytic activity. The maximal hemolysis of DLP2 and DLP4 was 2.31% and 1.46% respectively at a high concentration of 512 μg/ml, which much lower than that of nisin (18.66 ± 3.43%) (Fig. 3G). Meanwhile, similar to vancomycin (7.09%), DLP2 showed slight inhibition of the activity towards the mouse peritoneal macrophages RAW264.7 cells (7.85%) at 128 μg/ml (Fig. 3H). Comparably, DLP4 showed a moderate cytotoxicity (17.39%). This suggested that DLP2 and DLP4 have low cytotoxicity against eukaryotic cells.

After 30 serial passages in the presence of peptides or antibiotics, the MICs of DLP2 and DLP4 against MRSA ATCC43300 did not change, which indicated that no mutants of bacteria resistant to peptides were produced (Fig. 3I). In contrast, both ceftriaxone and ciprofloxacin induced obvious bacterial resistances with the MICs increased by 256- and 4-fold, respectively. These features of DLP2 and DLP4 indicate that the two peptides may serve as good candidates for the development of novel antibacterial agents against resistant bacteria.

### Antibacterial mechanism analysis of DLP2 and DLP4 against *S*. *aureus*

#### Permeabilization of the bacterial plasma membrane

**Figure 4:**
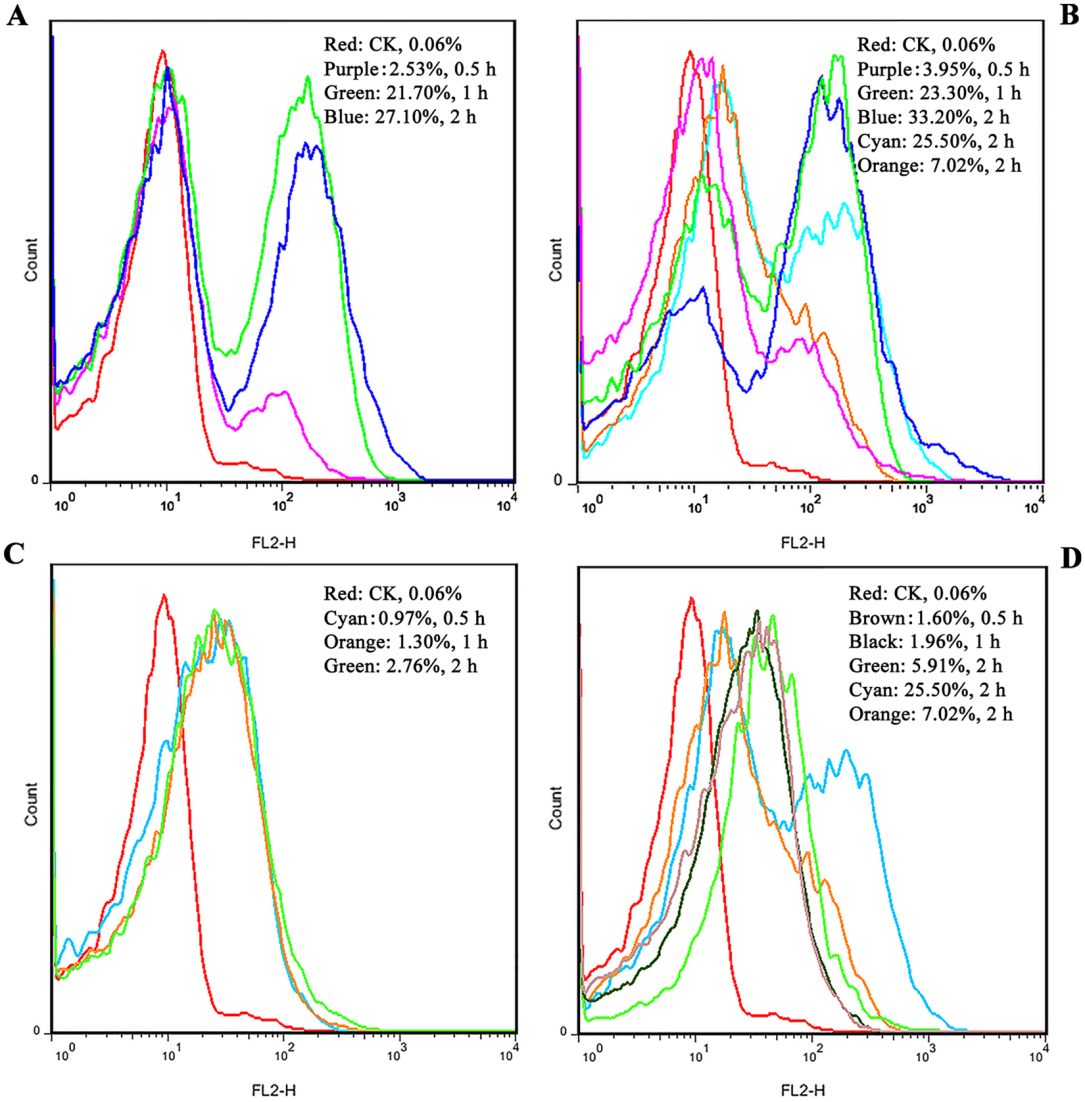
FACS analysis of PI staining in MRSA ATCC43300. (**A**,**B**) Cells treated with 2× (**A**) and 4× MIC (**B**) DLP2 or antibiotics, respectively. Red line: negative control; purple, green, and blue lines: treatment with peptides for 0.5, 1, and 2 h, respectively; cyan and orange lines: treatment with 4× MIC bacitracinis and vancomycin for 2 h, respectively. (**C**) Cells treated with 2× MIC DLP4. Red line: negative control; cyan, orange and green lines: treatment for 0.5, 1, and 2 h, respectively. (**D**) Cells treated with 4× MIC DLP4 or antibiotics. Red line: negative control; brown, black and green lines: treatment with DLP4 for 0.5, 1, and 2 h, respectively; cyan and orange lines: treatment with bacitracinis and vancomycin for 2 h, respectively.

A fluorescence dye propidium iodide (PI), which can penetrate the damaged cell membrane and intercalate into nucleic acid, was used as an indicator to evaluate effects of DLP2 and DLP4 on the plasma membrane of MRSA ATCC43300 cells by flow cytometry^18^. As shown in Fig. 4, in the absence of peptides, the percentage of *S*. *aureus* stained with PI was 0.06%, indicating the integrated cell membranes. The percentages of PI-positive MRSA ATCC43300 cells treated with 2 × MIC DLP2 for 0.5 h, 1 h and 2 h were 2.53%, 21.70% and 27.10%, respectively (Fig. 4A). After treatment with 4 × MIC DLP2 for 0.5 h, 1 h and 2 h, the percentages of *S*. *aureus* cells stained with PI were 3.95%, 23.30% and 33.20%, respectively (Fig. 4B). Comparably, the percentages of PI-positive cells treated with 4 × MIC bacitracin and vancomycin for 2 h were 25.5% and 7.02% respectively, which is even lower than those of cells treated with 2 × MIC DLP2 (27.1%) but largely higher than ones treated with DLP4 (2.76‒5.91%) (Fig. 4A,C and D). This suggested that the plasma membrane of *S*. *aureus* may be potently disrupted by DLP2 in a time- and concentration- dependent manner, but weakly damaged by DLP4.

#### Inducing ultrastructural change in cells

**Figure 5:**
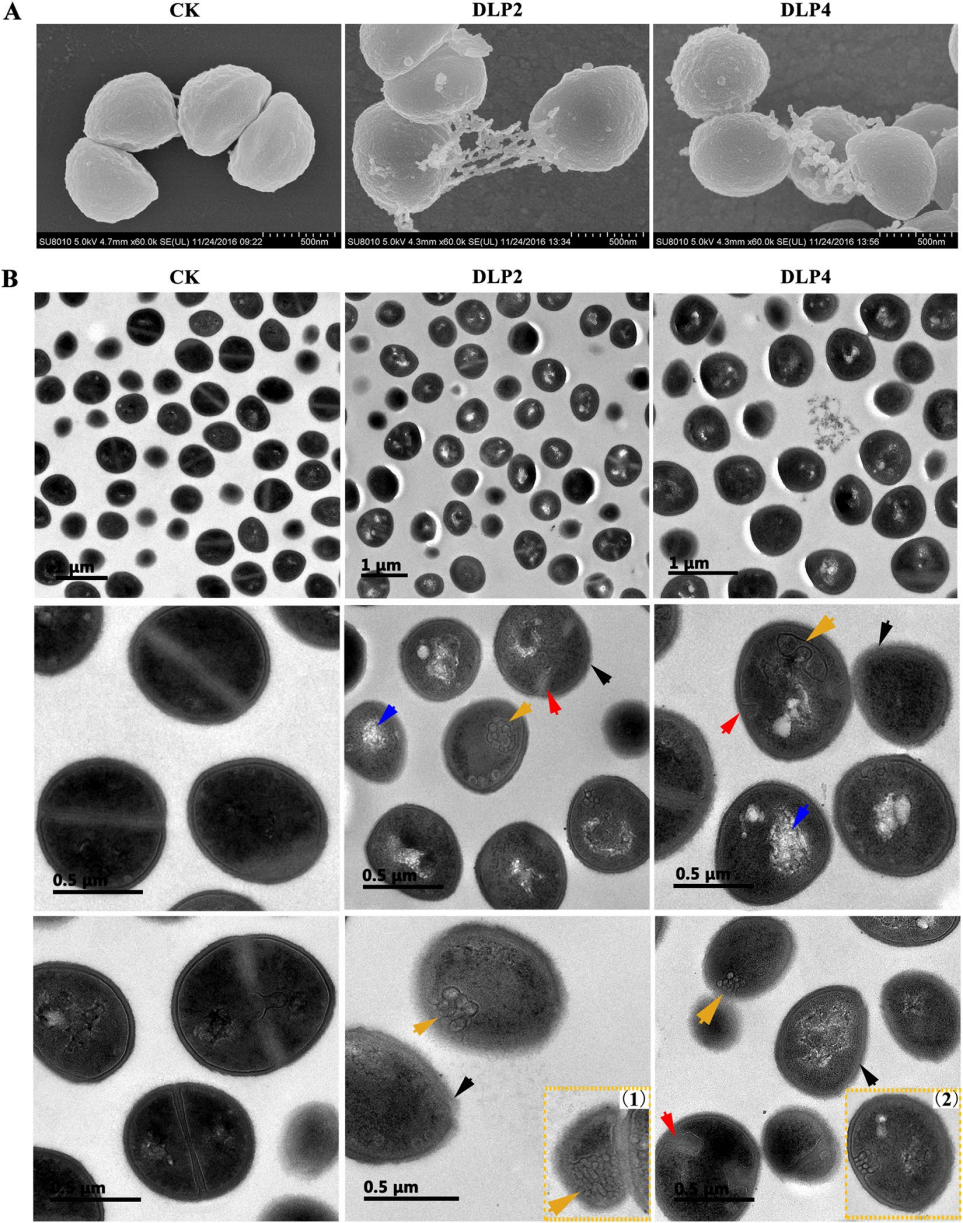
Effects of DLP2 and DLP4 on the cell morphology and ultrastructure of MRSA ATCC43300. Bacteria in mid-logarithmic growth were treated with peptides at 2× MIC for 2 h. (**A**) SEM images. (**B**) TEM images. Red arrows: irregular smallish septa; yellow arrows: mesosome-like structures (1, 2); blue arrows: electron-light regions; black arrows: thinned cell wall.

Scanning electron microscopy (SEM) and transmission electron microscopy (TEM) were used to directly observe the effects of DLP2 or DLP4 treatment on the cell morphology and integrity of MRSA ATCC 43300. As shown in Fig. 5, the untreated *S*. *aureus* cells exhibited the smooth surface and intact cell morphology with a prominent septal midline. After exposure of *S*. *aureus* to 2 × MIC DLP2 or DLP4 for 2 h, some filiferous adhesions were observed outside of cells (Fig. 5A). The TEM images showed that bubble flow mesosome-like structures were gathered together and observed in the DLP2- and DLP4-treated *S*. *aureus* cells but not in the untreated control cells, which acting as a defensive action of bacteria. Deformed smallish septa, disappeared cytoplasmic membrane, heterogeneous electron density, electron-light region and thinned cell wall were also observed in treated *S*. *aureus* cells (Fig. 5B). Approximately 33.3%, 20% and 9.9% cell division cells were observed in control group, DLP2-treated and DLP4-treated groups respectively, indicating that the action of two peptides maybe suppress cell division process^19^.

#### Binding to bacterial genomic DNA

**Figure 6:**
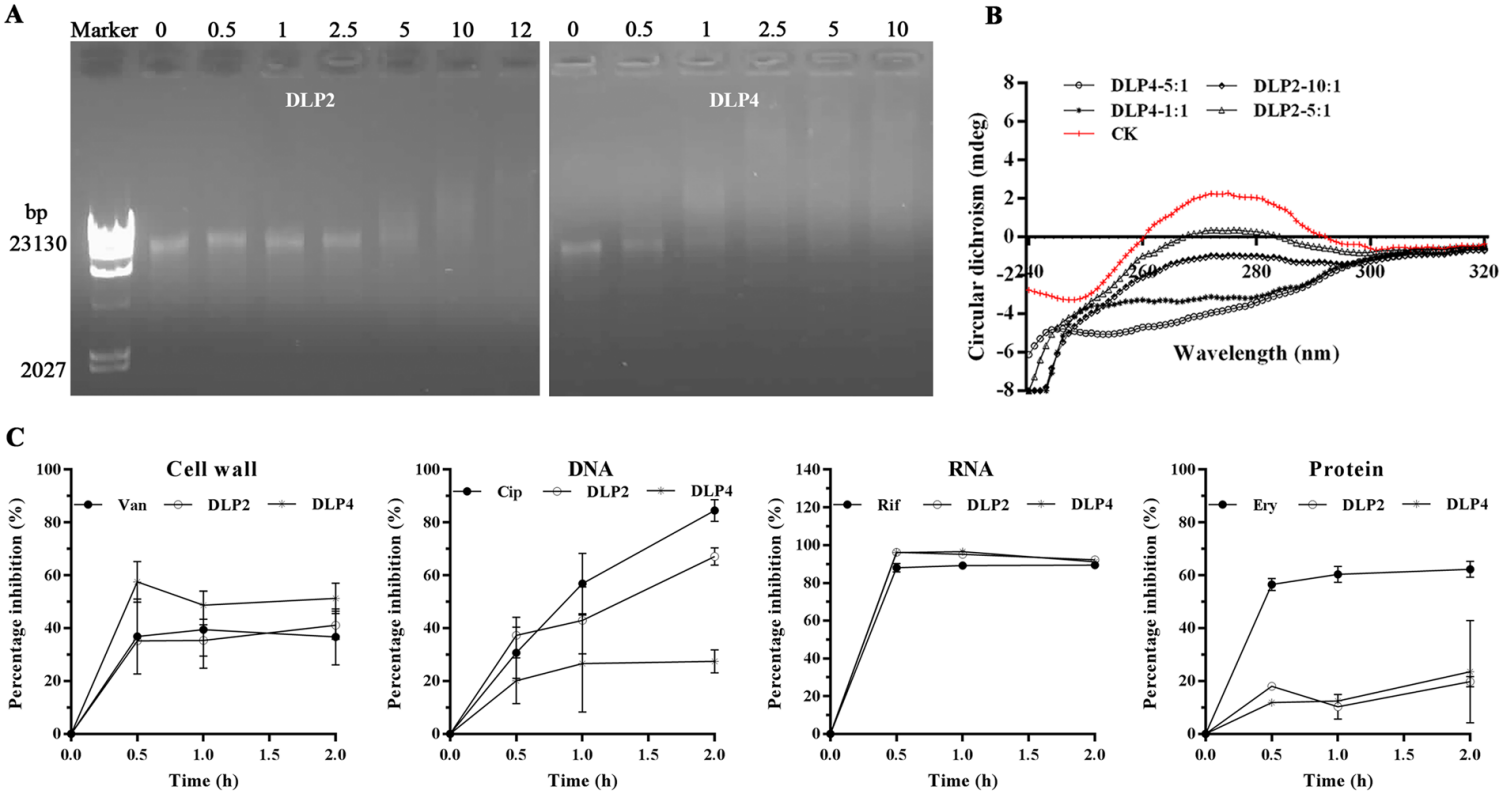
DLP2 and DLP4 binding to bacterial genomic DNA and their effects on macromolecular synthesis. (**A**) Gel retardation analysis of binding of DLP2 and DLP4 to DNA. Marker, λ *hind*III. Lanes 2 to 8, the mass ratios of DLP2 and genomic DNA from MRSA ATCC43300 were 0, 0.5, 1, 2.5, 5, 10, and 12, respectively. Lanes 9 to 14, the mass ratios of DLP4 and genomic DNA from *S*. *aureus* were 0, 0.5, 1, 2.5, 5, and 10, respectively. The full-length gels are presented in Supplementary Figure 8. (**B**) The CD spectra of genomic DNA from *S*. *aureus*, mass ratios of peptides and DNA were 1, 5 and 10, respectively. (**C**) Inhibition of cell wall, DNA, RNA and protein synthesis in *S*. *aureus* by 2× MIC DLP2 and DLP4. Vancomycin (Van, 2× MIC), ciprofloxacin (Cip, 8× MIC), rifampicin (Rif, 4× MIC) and erythrocin (Ery, 2× MIC) were used as controls. Results were given as mean ± SD (n = 3).

To seek the potential intracellular targets, the DNA-binding ability of DLP2 and DLP4 was evaluated by the DNA gel retardation assay. As shown in Fig. 6A, both DLP2 and DLP4 didn’t disturb the migration of the genomic DNA from MRSA ATCC43300 at the peptide/DNA mass ratios lower than 5 and 1, respectively. However, at the mass ratio over 5 and 1, all DNA were dispersive in gel lane and no DNA bands were detected for DLP2 and DLP4 on the gel, indicating that DNA-binding activity of DLP4 is higher than that of DLP2, which may be related to lower hydrophobicity of DLP4^20^. In contrast, DLP2 and DLP4 weakly bound to the genomic DNA from *E*. *coli* CVCC1515 and *S*. *typhimurium* ATCC14028 (Supplementary Fig. 5S), indicating the ability of DLP2 and DLP4 to bind bacterial DNA non-specifically. The intrinsic DNA-binding ability of DLP2 and DLP4 were further supported by the following CD spectroscopy.

As shown in Fig. 6B, right-handed B-form DNA from MRSA ATCC43300 was observed in the absence of peptides, which was characterized by a negative band at approximately 250 nm and a positive band at approximately 275 nm. Compared to DLP4, there was a smaller decrease in the CD amplitude for DLP2 but no obvious shift, suggesting that DLP2 damaged DNA helical structure and weakened its base stacking force (Fig. 6B). However, the CD spectra were dramatically changed when DLP4 bound to DNA from *S*. *aureus*, indicating that DLP4 maybe alter DNA conformation^21^.

#### Arresting cell cycle progression and inhibiting DNA synthesis

The effects of DLP2 and DLP4 on cell cycle progression of *S*. *aureus* were investigated using flow cytometry. The percentages of the untreated cells in I-, R-, and D-phase were 15.39%, 78.2% and 6.4%, respectively (Fig. S3A). Exposure to 2 × MIC DLP2 or DLP4 for 0.5 h or 2 h resulted in a reduction in the percentages of cells in the I- (from 9.42% to 7.68%, from 11.24% to 9.9%) and D-phase (from 5.25% to 3.83%, from 6.33% to 6.24%) and an increase in percentage of R-phase cells (from 85.33% to 88.48%, from 82.43% to 83.86%) in a time-dependent manner (Supplementary Fig. S3B,C,E and F). The percentages of ciprofloxacin-treated cells in I-, R- and S- phase for 0.5‒2 h were 11.57‒7.27%, 86.04‒90.76%, and 2.45‒1.97%, respectively (some data not shown; Supplementary Fig. S3D). The results suggested that similar to ciprofloxacin, DLP2 and DLP4 induced the cell cycle of *S*. *aureus* arrested at R-phase and the two peptides maybe inhibit the replication of DNA.

The incorporation of radioactive precursors into DNA, RNA, cell wall and protein was measured to demonstrate the effects of DLP2/DLP4 on macromolecular synthesis in MRSA ATCC43300. As shown in Fig. 6C, a significant inhibition of ^3^H-uridine (92.23‒96.21% and 91.69‒96.55%, respectively) and D-[6-^3^H(N)] glucosamine hydrochloride (35.25‒41.13% and 48.74‒57.57%, respectively) incorporation was observed at 0.5 h of exposure of *S*. *aureus* to 2 × MIC DLP2 or DLP4. Moreover, both DLP2 and DLP4 also induced a weak decrease in DNA synthesis (37.28‒67.14% and 20.08‒27.46%, respectively) in a time-dependent manner and protein synthesis (10.30‒19.77% and 11.89‒23.57%, respectively) (Fig. 6C), which was similar to P-Der^22^. This suggested that DLP2 and DLP4 may be inhibitors of RNA, cell wall and DNA synthesis, which is similar to rifampicin, vancomycin and ciprofloxacin, respectively.

### DLP2 and DLP4 protected mice from a challenge with MRSA ATCC43300

#### Protection of mice against a lethal bacterial challenge

**Figure 7:**
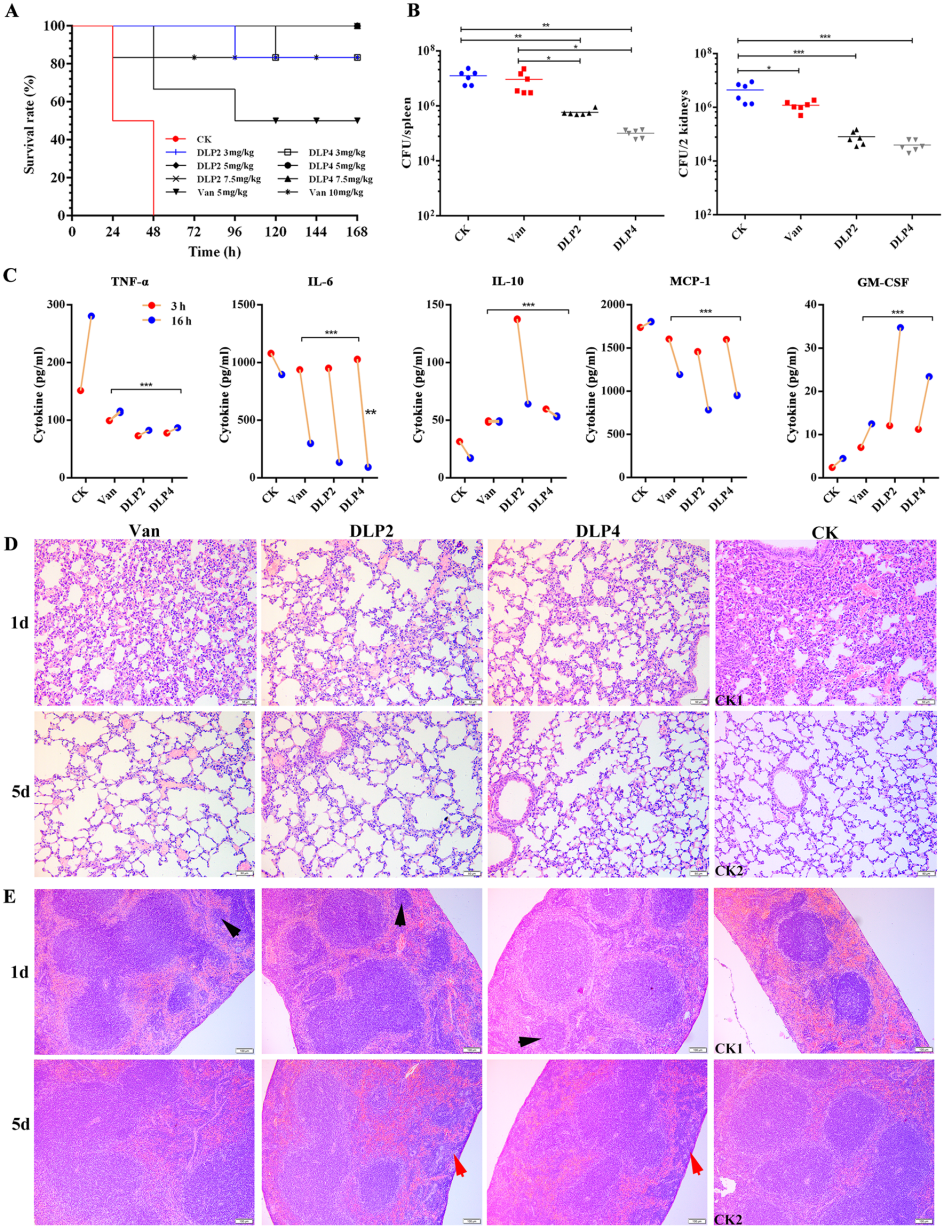
Protection efficacy in bacterial infection mice models. (**A**) Survival of mice. Mice were infected intraperitoneally with MRSA ATCC43300 (1 × 10^10^ CFU) and treated intraperitoneally with DLP2, DLP4, and vancomycin at 2 h and 12 h after post-infection, respectively. Survival of mice was recorded for seven days. (**B**) Effect of peptides on splenic and nephritic bacterial burdens in *S*. *aureus*-infected mice. Mice were infected intraperitoneally with MRSA ATCC43300 (8 × 10^9^ CFU) and treated with a single dose of DLP2, DLP4 (7.5 mg/kg), and vancomycin (10 mg/kg) at 2 h post infection, respectively. Kidneys and spleens were removed at 3 h post-treatment to analyze bacterial translocation. Data are expressed as mean ± standard deviation. ^*^p < 0.05; ^**^p < 0.01; ^***^p < 0.001. Statistical significance of differences between experimental groups of animals was determined using the one-way ANOVA and Tukey multiple comparison. Data points represent the number of CFU from the indicated organ of individual mice; horizontal bars indicate the mean CFU values for each group. (**C**–**E**) Effects of DLP2 and DLP4 on sera cytokines and organ injury. Mice were infected intraperitoneally with MRSA ATCC43300 (8 × 10^9^ CFU) and treated with DLP2, DLP4 (7.5 mg/kg), and vancomycin (10 mg/kg). Sera were collected and cytokines were detected at 3 and 16 h after treatment, respectively. Lungs and spleens were harvested and detected at 1 d, 3 d and 5 d post-infection. Statistical analysis was performed using the one-way ANOVA and Bonferroni multiple comparison. ^**^p < 0.01; ^***^p < 0.001. Vancomycin and PBS served as positive and negative controls for all experiments. (**C**) Sera TNF-α, IL-6, IL-10, GM-CSF, and MCP-1 levels. (**D**) Lung tissues. (**E**) Spleen tissues. (**D**,**E**) CK1: the infected mice without treatment; CK2: the uninfected mice.

To evaluate the therapeutic activity of peptides in the peritonitis model, mice were challenged with *S*. *aureus* (1 × 10^10^ CFU) and followed by treatment with DLP2 and DLP4. As shown in Fig. 7A, the untreated mice began to die at 12 h after inoculation with *S*. *aureus*, and all were dead within 36 h. Mice injected with PBS in the control group survived throughout the experimental period. After treatment with 3, 5 and 7.5 mg/kg DLP2 or DLP4, the survival rates of mice were 80%, 100% and 100%, respectively. In the positive control, the survival rates of mice treated with 5 and 10 mg/kg vancomycin were 60% and 83.3% respectively, which was lower than DLP2 and DLP4. This result indicated that both DLP2 and DLP4 can protect mice from a lethal *S*. *aureus* challenge *in vivo*.

#### Inhibition of bacterial translocation

To test whether intraperitoneal inoculation (8 × 10^9^ CFU) leads to the translocation of *S*. *aureus* into deep organs, infected mice were treated with DLP2, DLP4 or vancomycin at 2 h post-inoculation, and spleen and two kidneys were harvested at 3 h post-treatment and analyzed by the plate count method. As shown in Fig. 7B, the number of viable *S*. *aureus* cells in spleen and two kidneys in mice treated with 7.5 mg/kg DLP2 or DLP4 decreased by 95‒99% and 98‒99%, respectively. Treatment with 10 mg/kg vancomycin resulted in a reduced bacterial burden in the spleen and two kidneys by 26% and 73%, respectively.

#### Inhibition of proinflammatory cytokines and stimulation of anti-flammatory cytokines

In parallel to translocation experiments, the serum levels of proinflammatory cytokines such as interleukin-6 (IL-6), interleukin-6I (IL-10), tumor-necrosis factor alpha (TNF-α), monocyte chemoattractant protein-1 (MCP-1) and granulocyte-macrophage colony-stimulating factor (GM-CSF) were determined at 3 h and 16 h post-treatment using an enzyme-linked immunosorbent assay (ELISA) kit. As shown in Fig. 7C, after 3 h treatment with DLP2, the concentrations of serum TNF-α, IL-6, IL-10, MCP-1 and GM-CSF of infected mice were 73.12‒82.46, 951.52‒136.16, 137.65‒64.08, 1460.37‒782.76, and 12.08‒34.75 pg/ml respectively and they were 77.79‒87.13, 1027.23‒93.31, 59.8‒53.37, 1602.02‒951.47, and 11.24‒23.42 pg/ml, respectively for mice treated with DLP4. The levels of TNF-α and MCP-1 in treated mice were significantly lower than those from the corresponding control groups (151.61‒280.57 and 1738.56‒1805.26 pg/ml, respectively) and the vancomycin groups (99.28‒115.17 and 1605.55‒1195.13 pg/ml, respectively). However, the levels of IL-10 and GM-CSF were significantly higher than those from the corresponding control groups (31.66‒17.23 and 2.42‒4.52 pg/ml, respectively) and the vancomycin groups (48.94‒48.94 and 7.04‒12.5 pg/ml, respectively). These data suggest that both DLP2 and DLP4 inhibit the production of proinflammatory cytokines TNF-α, IL-6 and MCP-1 but stimulate the synthesis of anti-inflammatory cytokines IL-10 and GM-CSF.

#### Suppression of acute lung and spleen injury

To explore if DLP2 and DLP4 can reduce lung and spleen injury from a lethal challenge with *S*. *aureus*, the organ damage degree was examined at 1 d and 5 d after treatment. As shown in Fig. 7D and E, no pathological change was observed in both lung and spleen of mice in the control group, whereas infected mice without treatment developed acute injury to a certain degree, and it was characterized by hyperemia and edema in interstitial, marked focal hemorrhage and inflammatory cells in alveolar space and neutrophils in lung, and by spleen atrophied, red pulp hemorrhage, and demolishing splenic corpuscles in spleen. In contrast, after treatment with DLP2 and DLP4, the lungs and spleens of the mice were apparently less damaged at 1 d, especially at 5 d post-treatment. The efficacy of both DLP2 and DLP4 (7.5 mg/kg) was higher than that of vancomycin (10 mg/kg), and DLP4 was superior to DLP2. Moreover, the increasing macrophages at the edge of the spleen were observed in mice only treated with DLP2 or DLP4, and many splenic corpuscles were developed in mice treated with the two peptides and vancomycin.

These data indicated that, superior to vancomycin, both DLP2 and DLP4 could protect mice from lethal *S*. *aureus* challenge *in vivo*.

## Discussion

The continuous and selective antibiotic pressure in humans and in farm animals have promoted the emergence of MRSA^23^, so more attentions have been concerned on the newly effective AMPs which have been considered as promising antibiotic alternatives in the medical field^6, 24^. In the current study, DLP2 and DLP4, the novel insect AMPs from *H*. *illucens*, were firstly expressed in *P*. *pastoris* and purified by cation-exchange columns; their antibacterial, pharmacological activities and the mechanism of action against MRSA ATCC43300 were systematically investigated for the first time.

Unlike the insect-derived Pro-rich non-CSαβ AMPs including abaecin, oncocin, apidaecin, Apidaecin 1b and Api88, which are predominantly active against Gram-negative bacteria such as *E*. *coli*, *Klebsiella pneumoniae*, and *P*. *aeruginosa*, insect CSαβ AMPs such as sapecin and lucifensin can inhibit the growth of Gram-positive bacteria (i.e. *S*. *aureus*)^25–27^. In the present study, however, CSαβ DLP2 and DLP4 exhibited a more potent antibacterial activity against Gram-positive bacteria than that found in the previous study^13^. DLP4 exhibited 2-‒4-fold higher antimicrobial activity compared to DLP2 (Table 2). Meanwhile, DLP4 could kill above 99% MRSA ATCC43300 within 2 h, indicating that it is more effective than DLP2 which needing at least 6 h (Fig. 3A). This may be directly attributed to higher positive net charge of DLP4 (+6) than DLP2 (+2) (Table 1), which is consistent with the previous studies which found that an increase in net charges leads to an enhancement of activity of CSαβ AMPs such as MP1106 against *S*. *aureus* ^28^. It has been demonstrated that positive charge is one of key factors in structure and antimicrobial activity of AMPs, and the large number of positively charged residues enable them to interact electrostatically with negatively charged cell surface molecules^29^. Moreover, positive net charge can also enhance interaction with anionic lipids and other bacterial targets^30^. Present results have demonstrated that mul-macromolecules are target sites for DLP2 and DLP4. The DNA-binding activity of DLP4 is higher than that of DLP2, which may also be related to weaker hydrophobicity of DLP4 (Table 1)^20^. However, the inhibition induced by DLP4 on DNA synthesis in *S*. *aureus* cells was lower than that by DLP2 (Fig. 6), which may be related to more potent membrane permeabilization of DLP2 than that of DLP4 (Fig. 4). Meanwhile, *in vivo* mice experiments, treatment with DLP4 (7.5 mg/kg) is more effective at reducing the *S*. *aureus* burden than treatment with DLP2 (7.5 mg/kg) and vancomycin (10 mg/kg) (Fig. 7B), which may be due to faster bactericidal efficacy and a significantly longer PAE of DLP4 with the exception of positive net charges (Fig. 3A and G)^31^.

PAE is an important pharmacodynamic parameter that may be considered in choosing the antibiotic dosing regiments in clinical use^31^. Results of the present study showed that both DLP2 and DLP4 obtained a significant concentration-dependent PAE, and shorter PAE was observed for MRSA ATCC43300 after exposure to DLP2 (0.67‒10.67 h) than DLP4 (3.33‒13.33 h) (Fig. 3C), which was 1.8-‒3.15-fold longer PAE, than vancomycin (2 h) in this study, NZ2114 (1.7‒3.5 h)^17^, and other antibiotics such as daptomycin (2 h), tigecycline (3.2 h), and arbekacin (3.0‒3.2 h) against *S*. *aureus* reported in previous studies^31–33^. Long PAEs might lead to longer interval of administration, which thus potentially reducing treatment cost and inhibiting development of drug resistance^34^.

It has been demonstrated that lucifensin and sapecin can permeabilize the cell membrane and form pores by formation of oligomers, which is based on electrostatic interaction between Asp4 of one peptide molecule and Arg23 of another peptide molecule because these two residues are situated at opposite ends of the oligomerization site^16^. Furthermore, both Asp4 and Arg23 residues are also conserved in lucifensin and insect defensins (Fig. 1A and C) and lead to membrane pore formation, but not for the absence of the Asp4 residue in truncated lucifensin^35, 36^. This suggests that the two charged residues Asp4 and Arg23 were play a vital role in the process of pore forming in cell membrane. On the contrary, however, both Asp4 and Arg23 simultaneously exist in DLP4, but not in DLP2, and led to weaker penetration the plasma membranes of *S*. *aureus* (Fig. 4). It may be related to Arg25 and Arg26 in DLP2, which attributed to a more hydrophobic α-helix (Supplementary Fig. S4E and F) and insertion deeper into the phospholipid membrane^37^.

Mesosomes, as intracytoplasmic membrane inclusions or invagination of the plasma membrane, are indicative of cytoplasmic membrane alteration, which have been regarded as structural artifacts in bacterial cells induced by antibiotics such as vancomycin, ciprofloxacin, gentamicin^38^, trimethoprim^39^, rifampin^40^ and cationic AMPs such as defensins^41^ and CP11CN^42^. Similarly, both DLP2 and DLP4 induced bubble flow mesosome-like structures in most *S*. *aureus* cells (Fig. 5B), indicating cytoplasmic membrane alteration and uncoupling of the synthesis and turnover of cell wall polymers^42^. We speculated that DLP2 and DLP4 might concentrate the oxidative phosphorylation enzymes and make the plasma membrane of the bacteria more fluid to form the mesosomes^38^. More interestingly, the mesosomes-like structures may be involved in several fundamental processes, including chromosome replication and segregation, exoenzyme transport, cell wall synthesis, and cell division^43^.

**Figure 8:**
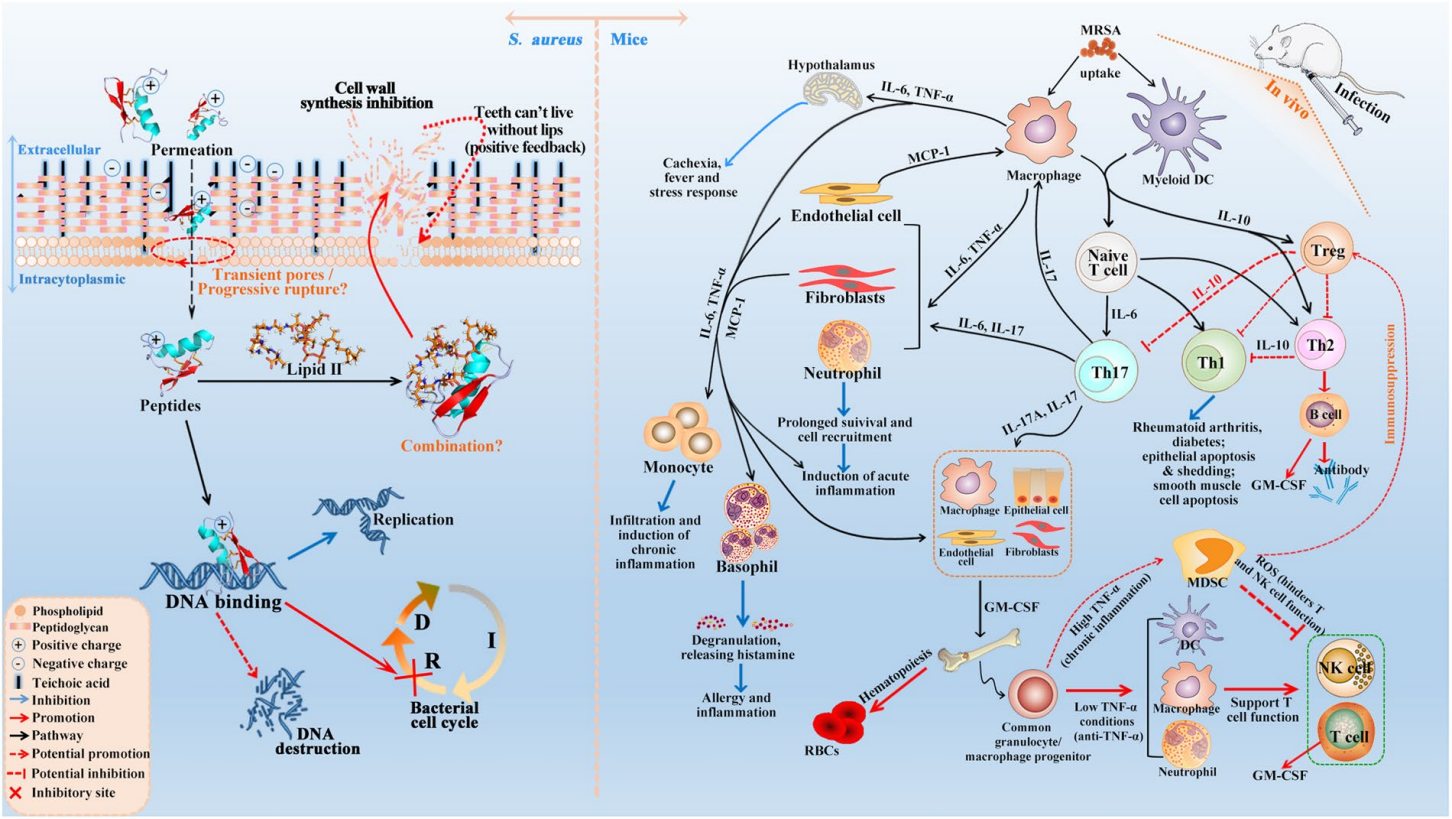
Proposed mode of actions of DLP2 and DLP4 against MRSA and their immunomodulatory pathways in mice. Left: DLP2 and DLP4 can penetrate plasma membrane, bind to DNA, induce cell cycle arrest at R-phase, inhibit DNA and cell wall synthesis, and form mesosome-like structures in MRSA cells. Right: DLP2 and DLP4 reduced serum pro-inflammatory cytokines levels (TNF-α, IL-6 and MCP-1) and promoted anti-inflammatory cytokines levels (IL-10 and GM-CSF). After stimulation by MRSA, macrophages and dendritic cell (DC) can produce cytokines, such as IL-6 and TNF-α, which in turn can activate neighboring cells such as endothelial cells and fibroblasts^46, 47^. This leads to formation of IL-6, TNF-α and MCP-1, that can then act locally, either systemically on induction of acute inflammation, or on monocytes or basophils^48^; therefore, reduction the release of these inflammatory factors caused by DLP2 and DLP4 may result in the infiltration of the former and induction of potential chronic inflammation, and the prolonged survival and degranulation of the latter and potential allergy and inflammation^46, 47, 49, 50^. Meanwhile, MRSA promote the development of T helper (Th) cells. An increase in IL-10 induced by DLP2 and DLP4 can mediate Th1 inhibition and promote antibody production^51, 52^. Activated B cells, T cells and the Th17 response can induce GM-CSF, in turn, leading to the mobilization of macrophages, neutrophils and eosinophils, as well as dendritic-cell maturation^53^. However, when a chronic inflammation happened (sustained high level of TNF-α), it can result in the accumulation of myeloid-derived suppressor cells (MDSCs), which then migrate to secondary lymphoid organs and tissues (such as the tumor site) and exert effects on other cell population. For example, induction of Tregs, indirect inhibition of immune response and inhibition of NK cell mediated innate immunity and the T cell suppression^54, 55^.

The pattern of macromolecular synthesis in the presence of DLP2 and DLP4 at its 2 × MIC appeared to be similar to that observed in the presence of the RNA polymerase inhibitor rifampin. This is also firstly evidenced by the present results that both DLP2 and DLP4 maybe inhibit cell wall biosynthesis in *S*. *aureus* (Fig. 6C), which is similar to lucifensin^44^. The high sequence similarity of these peptides (>60%) (Fig. 1A) suggests that DLP2 and DLP4 maybe have the same mode of action as lucifensin^45^, including binding to the cell wall precursor-lipid II, permealization of cell membrane, and formation of membrane pore formation (Fig. 8). Secondly, it is demonstrated by our another result that DLP2 and DLP4 prevented cells from entering the D-phase of the cell cycle, resulting in the accumulation of cells in R-phase where DNA replication occurs (Supplementary Fig. S3). Moreover, after treatment with DLP2, the proportion of R-phase cells was higher than that of DLP4, indicating more potent ability of DLP2 than DLP4, which is consistent with the result of membrane permealization (Fig. 4). This possible multiple mode of action of DLP2 and DLP4 makes it more difficult for *S*. *aureus* to develop resistance within 30 days (Fig. 3I). Comparably, however, common antibiotics, such as ciprofloxacin and cefatriaxone induced a 4-‒256-fold increase in MIC (Fig. 3I), which may be related to their different mechanism of action, which needing further study.

The study of immunomodulation activities of DLP2 and DLP4 on mice infected with MRSA showed that DLP2 is more effective to inhibit *S*. *aureus*-evoked expression of pro-inflammatory cytokines of TNF-α, IL-6 and MCP-1, and promote production of anti-inflammatory ones of IL-10 and GM-CSF than DLP4 and vancomycin (Fig. 7C). TNF-α and IL-6, which have been considered the primary mediators of sepsis, can act synergistically for the development of fever and induce septic shock by vascular permeability, hemorrhage and severe pulmonary edema^43^. Moreover, TNF-α is known to cause further inflammatory reactions via stimulating the production of cytokines or chemokines including MCP-1^46^ (Fig. 8), one of the most potent chemoattractants for monocytes and lead to the emigration of monocytes into inflamed tissue^47^. These monocytes usually provide for a competent immune reaction, but excessive accumulation of monocytes may coincide with extensive tissue damage by mononuclear cell infiltration including atherosclerosis, rheumatoid arthritis, and multiple sclerosis^49^, mainly due to mediators released by activated monocytes plasma IL-6 levels in patients with sepsis, which are markedly increased, particularly in patients who develop a fatal septic shock^50^. During *S*. *aureus* infection, the bacteria activate TLR2/6 heterodimers, and TLR2 recruits myeloid differentiation factor 88 (MyD88) to activate the downstream signaling (Fig. 8). Then, TLR2-MyD88 signaling complex recruits TNF receptor-associated factor 6 (TRAF6) to mediate mitogen-activated protein kinases (MAPKs) and nuclear factor κB (NF-κB) activation, which account for the production of pro-inflammatory cytokines (TNF-α, IL-6, etc.) and anti-inflammatory cytokine of IL-10^51^. Anti-inflammatory cytokine of IL-10 plays a crucial role in limiting inflammatory responses and preventing host damage^52^. In our study, it is demonstrated that both DLP2 and DLP4 play a protective role against sepsis induced by MRSA ATCC43300 likely through down-regulating the expression of pro-inflammatory cytokines and up-regulating anti-inflammatory cytokines levels (Fig. 7), which is consistent with previous studies that blocking IL-6 and promoting IL-10 by ephedrine hydrochloride and micheliolide were reported to improve the survival of mice in sepsis^51, 56^. Taken together, both DLP2 and DLP4 maybe maintain the balance of pro-inflammatory and anti-inflammatory cytokines in response to MRSA ATCC43300, which make them promising candidates for the treatment of staphylococcal especially MRSA infections.

In this study, both DLP2 and DLP4 were successfully expressed in *P*. *pastoris* and showed a potent antimicrobial activity against Gram-positive bacteria especially MRSA. The two peptides demonstrated low hemolysis and cytotoxicity, no resistance, and greater potency and a significantly longer PAE than vancomycin. DLP2 and DLP4 disrupted the plasma membrane, induced cell cycle arrest at R-phase, and inhibited multi-macromolecular synthesis in MRSA (Fig. 8). Both DLP2 and DLP4 protected mice from mortality challenge with MRSA and inhibited inflammatory response (Fig. 8). Our results suggest that DLP2 and DLP4 are a promising class of potential antimicrobial candidates for the treatment of staphylococcal infections especially MRSA.

## Materials and Methods

### Structure analysis of DLP2 and DLP4

Multiple sequences alignment and the sequence conservation analysis were performed by ClustalX 1.8 and the WebLogo (http://weblogo.berkeley.edu/logo.cgi). The three-dimensional structures of DLP2/DLP4 were modeled using SWISS-MODEL workspace (http://swissmodel.expasy.org/workspace/) and PyMol 1.8.

### Expression, purification, and identification of DLP2 and DLP4

The construction of pPICDLP2 and pPICDLP4 plasmids was described in detail in the supplemental material. Positive transformants containing pPICDLP2 and pPICDLP4 plasmids were expressed at the shaking flask and fermenter level according to the previous protocol^28^.

### *In vitro* pharmacodynamics of DLP2 and DLP4 and their synergism with antibiotics

#### The MIC and time-kill curve

The MIC values of DLP2 and DLP4 against bacterial strains were determined in Mueller Hinton broth (MHB) (casein hydrolysate 17.5 g/l, soluble starch 1.5 g/l, and beef infusion solids 5 g/l, pH 7.0 ± 0.2) (AOBOX Biotechnology, China) using a broth microdilution technique as previously described^25^. A time-kill curve assay was used to evaluate the pharmacodynamics of DLP2 and DLP4 against MRSA ATCC43300. The methods were described in detail in the supplemental material.

#### Intracellular antibacterial activity

Murine macrophage RAW264.7 cells (2.5 × 10^5^) were incubated with mid-exponential-phase MRSA ATCC43300 for 2 h at 37 °C in a 5% CO_2_ incubator. Gentamicin (100 μg/ml) was added into the cultures and incubated for 1 h and then thoroughly washed with PBS for three times to remove extracellular bacteria^57^. Fresh DMEM containing different concentrations of DLP2, DLP4 or vancomycin (2, 10, and 20 × MIC) were added into the cultures and incubated at 37 °C for 18 h. Monolayers cells were then washed three times with PBS, lysed with lysis buffer (1% Triton X-100, 20 mM Tris, 0.2 M NaCl, 2 mM EDTA). The internal *S*. *aureus* cells were estimated by colony counting on MHA (casein hydrolysate 17.5 g/l, soluble starch 1.5 g/l, beef infusion solids 5 g/l, and agar 12.5 g, pH 7.0 ± 0.2) plates.

#### PAE

The PAE of DLP2 and DLP4 against MRSA ATCC43300 was determined in Muller Hinton (MH) by the viable plate count method as described previously^58^ in the supplemental material.

#### Synergism test

The interaction of combinations of peptides and traditional antibiotics was evaluated in MHB by a checkerboard microtiter assay as described in detail in the supplemental material and expressed as FICI for each agent^59^.

### Stability, toxicity, and resistance of DLP2 and DLP4

The thermal stability of DLP2 and DLP4 was determined after 1-h incubation of 25 μl (32 μg/ml) of peptides at 4, 20, 40, 60, 80, and 100 °C in deionized water, respectively. The antimicrobial activity of DLP2 and DLP4 against *S*. *aureus* strain ATCC25923 was tested using an inhibition zone assay^16^.

The hemolytic activity of DLP2 and DLP4 was evaluated against fresh mRBCs by determining the amount of the released hemoglobin at 540 nm^60^. The method was described in detail in the supplemental material.

The cytotoxicity of DLP2 and DLP4 on the viability of mouse peritoneal macrophages RAW264.7 cells was measured by a colorimetric 3-(4,5-dimethylthiazol-2-yl)-2,5-diphenyl-2H-tetrazolium bromide assay (MTT) according to the method in the supplemental material.

The resistance experiment for DLP2 and DLP4 was performed in MH by sequential passaging. These methods are described in detail in the supplemental material.

### Interaction of DLP2 and DLP4 with the *S*. *aureus* membrane

The membrane permeabilization activity of DLP2 and DLP4 was investigated by a PI uptake assay as described in the supplemental material.

### SEM and TEM

The exponential-phase MRSA ATCC43300 were treated with 2 × MIC DLP2 or DLP4 for 2 h at 37 °C, fixed with osmium tetroxide, dehydrated by ethanol, and CO_2_ dried. The samples were sputter-coated with platinum and observed using a QUANTA200 SEM (FEI, Philips, Netherlands) as described in detail in the supplemental material.

After treatment with peptides for 2 h, the MRSA ATCC43300 cells were fixed twice, dehydrated with ethanol, embedded in Spur resin, and sectioned. Then, samples were stained with uranyl acetate and lead citrate and observed using a TEM (JEM-1400, JEDL, Tokyo, Japan) as described in the supplemental material.

### Interaction of DLP2 and DLP4 with *S*. *aureus* DNA

To examine whether binding of DLP2 and DLP4 to bacterial DNA, the gel retardation experiment was performed by mixing peptides and bacterial DNA, which was extracted from MRSA ATCC43300 using a TIANamp Bacteria DNA Kit (TIANGEN Biotech Co., Ltd., Beijing) as described in detail in the supplemental material.

CD measurements were also carried out on a MOS-450 spectropolarimeter using a quartz cuvette with 1.0-mm path length to examine further DLP2 and DLP4 binding to DNA at the ratios of 1, 5 and 10, respectively. The spectra were the average of 10 scans collected from 220 to 320 nm at 25 °C with a 20 s bandwidth.

### Effect of DLP2 and DLP4 on the cell cycle of *S*. *aureus*

The cell cycle of *S*. *aureus* treated with DLP2 and DLP4 was analyzed by PI staining using flow cytometry according to a previous method in the supplemental material^60^.

### Effect of DLP2 and DLP4 on the macromolecular synthesis of *S*. *aureus*

Effect of DLP2 and DLP4 on the macromolecular synthesis of *S*. *aureus* was measured by isotope labeling experiment as described in detail in the supplemental material.

### Mouse *in vivo* experiments

All mouse experiments were performed in accordance with the Animal Care and Use Committee of the Feed Research Institute of Chinese Academy of Agricultural Sciences (CAAS) and protocols were approved by the Laboratory Animal Ethical Committee and its Inspection of the Feed Research Institute of CAAS (AEC-CAAS-20090609).

To establish a peritonitis or sepsis mouse model, six-week-old female BALB/c mice (five mice/group) were intraperitoneally injected with MRSA ATCC43300 (2 × 10^10^ CUF/ml, 0.5 ml) at the lower left side of abdomen, and followed by intraperitoneal injection at other side with two doses of DLP2, DLP4 (3, 5, and 7.5 mg/kg of body weight, 0.3 ml) or vancomycin at 2 h and 12 h post-infection, respectively. Mice injected with only bacteria or PBS were used as negative or blank controls. Survival of mice was recorded daily for seven days.

Similarly, mice (30 mice/group) were intraperitoneally injected with MRSA ATCC43300 (8 × 10^9^ CUF/ml, 0.5 ml) and treated with a single dose of DLP2, DLP4 (7.5 mg/kg) or vancomycin (10 mg/kg) at 2 h post infection. Sera was collected from the mice sacrificed at 3, and 16 h post-treatment. The levels of TNF-α, IL-6, IL-10, GM-CSF, and MCP-1 in sera were detected at Jiaxuan Biotech. Co. Ltd. (Beijing, China) using an ELISA kit^61, 62^. Moreover, kidneys and spleens were removed at 3 h post-treatment and homogenized in sterile PBS for a CFU assay for MRSA ATCC43300 to evaluate bacterial translocation.

Mice were intraperitoneally injected with DLP2, DLP4 (7.5 mg/kg) or vancomycin (10 mg/kg) at 2 h post-intraperitoneal infection of MRSA ATCC43300 (8 × 10^9^ CUF, 0.5 ml). Lungs and spleens were harvested from mice which were sacrificed at 1 d, 3 d and 5 d after infection, washed with PBS, and fixed in 4% paraformaldehyde at 4 °C for 24 h. After washing with PBS and dehydrating with a graded series of ethanol (75‒95%), the organs were infiltrated with xylene, embedded in paraffin wax and sectioned. After staining with hematoxylin and eosin, the tissue samples were examined by a light microscope. Mice treated with vancomycin or PBS served as a positive or negative control, respectively.

### Statistical analysis

All data are presented as means ± standard deviation (SD). GraphPad Prism software v6.0 (GraphPad Software, USA) was used for all statistical analyses.

## Electronic supplementary material

Supplementary Information
