# Supplementary material for: Antibacterial and immunomodulatory activities of insect defensins-DLP2 and DLP4 against multidrug-resistant Staphylococcus aureus
Source: Sci Rep. 2017 Sep 21;7:12124. doi: 10.1038/s41598-017-10839-4 (PMC5608901; doi:10.1038/s41598-017-10839-4)
Supplement: Supplementary file 1 — Supplementary Information [file 41598_2017_10839_MOESM1_ESM.pdf]

Antibacterial and immunomodulatory activities of insect defensins-DLP2 and DLP4 against multidrug-resistant *Staphylococcus aureus*

Zhanzhan Li[1, 2*], Ruoyu Mao[1, 2*], Da Teng[1, 2], Ya Hao[1, 2], Huixian Chen[1, 2], Xiumin Wang [1, 2], Xiao Wang[1, 2], Na Yang[1, 2], Jianhua Wang[1, 2**]**

SUPPORTING INFORMATION

Supplementary 1: Materials and Methods

Materials

Escherichia coli DH5α (Invitrogen, Beijing, China), *Pichia pastoris* X-33 (Invitrogen, Beijing, China), and pPICZαA vectors (Invitrogen, Beijing, China) were used for cloning and expression. Multidrug-resistant *Staphylococcus aureus* ATCC43300 (MRSA ATCC43300) and *S. aureus* ATCC25923 were used to assay the antibacterial activity of the purified peptides as representative of Gram-positive bacteria. Restriction enzymes *Eco*RI and *Xho*I and T4 DNA ligase were purchased from Promega (Promega, USA). The protein marker was purchased from TIANGEN (Tiangen, China). Vancomycin and gentamicin were purchased from the China Institute of Veterinary Drug Control. Triton X-100, propidium iodide (PI) were purchased from Sigma-Aldrich. All other chemical reagents used were of analytical grade.

Specific pathogen-free female BALB/c mice (6 weeks old) were purchased from Vital River Laboratories (VRL; Beijing, China).

Construction of the recombinant plasmid and screening for transformants

Both *DLP2* and *DLP4* genes were optimized using the Reverse Translate Tool (http://www.bioinformatics.org/sms2/rev_trans.html) based on the preferential codon usage of *P.*

pastoris (http://www.kazusa.or.jp/codon/). The two genes, containing two restriction enzyme recognition sites (*Xho*I and *Xba*I) and a yeast Kex2 protease cleavage site, were synthesized by Sangon Biotech (Shanghai, China) and amplified by PCR with the primers of 3′AOX (5′-GCAAATGGCATTCTGACATCC-3′) and 5′AOX (5′-GACTGGTTCCAATTGACAAGC-3′). The two genes were inserted into the pPICZαA plasmid between *Xho*I and *Xba*I sites and transformed into *E. coli* DH5α. The pPICDLP2, pPICDLP4 and pPICZαA (negative control) vectors were linearized with *Bgl*II and transformed into competent *P. pastoris* X-33 cells[1]. Positive transformants were selected on YPDS plates (10 g/l yeast extract, 20 g/l peptone, 20 g/l glucose, 1 M sorbitol, and 20 g/l agar) with 100 mg/l Zeocin and were further confirmed by PCR amplification using the primers F: 5′-CCGCTCGAGAAGAGAGGTTT-3′ and R: 5′-GCTCTAGATTATTAGTAACAC-3′ and by sequencing, respectively.

Expression of DLP2 and DLP4 at the shaking flask and fermenter level

Positive transformants were cultured at 29°C (250 rmp) in 20-ml BMGY medium (1% yeast extract, 2% peptone, 1% glycerol, 1.34% yeast nitrogen base, 0.004% biotin, 0.1 M PBS, pH 6.0) in 50-ml shaking flasks to an $OD_{600\ nm}$ of 5.0. After centrifugation at 2000 g for 5 min, cells were resuspended in BMMY medium (1% yeast extract, 2% peptone, 0.5% methanol, 1.34% yeast nitrogen base, 0.004% biotin, 0.1 M PBS, pH 6.0) to an $OD_{600\ nm}$ of 1.0. Methanol (0.5%) was added into cultures every 24 h during the 120-h induction period.

To improve the production of peptides, high-density fermentation was performed in a 5-l fermentor (BIOSTATB plus, Sartorius Stedim Biotech) as described previously[1]. Briefly, positive transformants were cultured at 29°C (250 rpm) overnight in 10 ml of YPD (100 μg/ml zeocin) in shaker flasks. Cultures were transferred into a 200-ml YPD medium, cultivated at 29°C (250 rpm) to an $OD_{600\ nm}$ of

6.0 and then inoculated into the 5-l fermentor, which contained 2-l basal salts medium (50 g/l $NH_4H_2PO_4$, 20 g/l K_2SO_4, 15 g/l $MgSO_4 \cdot 7H_2O$, 6 g/l KH_2PO_4, 0.4 g/l $CaSO_4$, and 1.5 g/l KOH). The temperature, stirring rate and aeration rate were controlled at 29°C, 1,000 rpm, and 8 l/min, respectively. Methanol was supplied from 1 to 6 ml/l/h within the first 6 h when the glucose was exhausted. The fermentation samples were taken every 12 h to quantify the wet cell weight. The secreted DLP peptide and total protein levels were preliminarily estimated using the inhibition zone assay against *S. aureus* (ATCC25923 and AT43300) and tricine-SDS−PAGE, respectively.

Purification and identification of DLP2 and DLP4

Fermentation supernatants were dialyzed using 1 kDa molecular weight cutoff dialysis tubing and freeze-dried, dissolved in 20 mM PBS buffer (pH 6.7) and purified by an SP sepharose FF cation-exchange column (length, 25 mm; internal diameter, 7 mm; GE Health-care), which was equilibrated with 20 mM PBS. After washing with binding buffer, protein samples were eluted with elution buffer (20 mM sodium phosphate buffer, 600 mM NaCl, pH 6.7). The eluent was analyzed by tricine-SDS–PAGE and inhibition zone assays[1]. The expressed peptides were identified by MALDI-TOF MS at the Laboratory of Proteomics, Institute of Biophysics, Chinese Academy of Sciences (CAS).

The fermentation supernatant was purified by an SP sepharose FF cation-exchange column and identification of DLP2 and DLP4 was confirmed by tricine-sodium dodecyl sulfate polyacrylamide gel electrophoresis (tricine-SDS–PAGE) and MALDI-TOF MS as in detail in the supplemental material.

The time-kill curve assay

The mid-log phase S. aureus cells (10^5 CFU/ml) were mixed with different concentrations of DLP2 and DLP4 (1, 2 and 4× MIC) and cultured at 37°C (200 rpm). A 100-μl sample was taken from the mixture

at an interval of 2 h, serially diluted and counted on plates. Vancomycin was used as a control.

Postantibiotic effect (PAE)

After exposure to DLP2, DLP4 (1 and 2× MIC) or vancomycin (2× MIC) for 2 h, *S. aureus* cells (107 CFU/ml) were diluted 1,000 times by fresh medium, transferred to new plates and incubated at 37°C with shaking at 250 rpm. The samples were taken from plates for counting every hour until bacterial cultures become turbid. Untreated bacteria were used as growth controls. The PAE was calculated using the equation: PAE = T − C, where T is time (hours) required for the CFU in the test culture to increase by 1 Log_{10} above the count immediately after dilution and C is the corresponding time (hours) for the growth control.

Synergism test

S. aureus cells (10^5 CFU/ml) were added into 96-well plates (100 μl/well), and incubated for 16~18 h at 37°C with a series of concentrations (0.125–8× MIC) of DLP2, DLP4 or antibiotics (ciprofloxacin, ceftriaxone sodium, kanamycin, vancomycin, and rifampicin). The following procedure was the same as that used for the MIC assay described above. FICI was calculated by the equation: FICI = FICA+ FICB = C_A^{comb} / MIC_A^{comb} + C_B^{comb} / MIC_B^{comb}, where MIC_A^{comb} and MIC_B^{comb} refer to the MICs of agents A and B when acting alone and C_A^{comb} and C_B^{comb} refer to concentrations of agents A and B at isoeffective combinations, respectively. The interaction between two agents was interpreted based on the FICI as follows: FICI > 2 indicates antagonism; 1 < FICI < 2, indifference; 0.5 < FICI≤ 1, additivity and FICI ≤ 0.5, synergy[2]. All experiments were performed in triplicate.

The toxicity and resistance of DLP2 and DLP4

The mRBCs were centrifuged at 1,500 rpm for 10 min at 4°C and washed with 0.9% NaCl for three times. The 8% mRBCs and DLP2/DLP4 solutions were added into 96-well plates. Maximum lysis

(100%) was determined by analyzing the supernatant of erythrocytes that had been incubated with 1% Triton X-100. Nisin and NaCl were used as the positive and negative control, respectively[3].

RAW264.7 cells (2.5× 10^4 cells/well) were seeded into 96-well and incubated at 37°C in the presence of 5% CO_2 for 24 h. After addition of peptides or antibiotics, the plate was incubated for 4 h. Supernatants were removed from the wells, the MTT dye was added into plates, and absorbance at 570 nm was determined on a microplate reader. Maximum cytotoxicity (100%) was determined by cells incubated with 0.1% TritonX-100, nisin and PBS were used as a control. The results were analyzed by the GraphPad Software using the Deming linear regression.

Resistance of DLP2 and DLP4 was performed by the MIC assays. The mid-log phase of MRSA ATCC 43300 (1× 10^5 CFU/ml) (90 μl/well) was added into 96-well plates. The solutions of DLP2, DLP4, ceftriaxone and ciprofloxacin (10 μl) were added into plates at concentrations of 8×, 4×, 2×, 1×, 0.5× and 0.25× MIC. The serial passaging was repeated for 30 d. After 18 h incubation at 37°C under continuous shaking at each passage, cells from the second highest concentration showing visible growth were used to inoculate the subsequent culture[4].

Interaction of DLP2 and DLP4 with the *S. aureus* membrane

The mid-log phase of MRSA ATCC43300 was harvested by centrifugation at 5,000 rpm for 5 min, washed with 0.01 M PBS (pH 7.4), and resuspended in the same buffer (1× 10^8 CFU/ml). Cells were incubated with or without 1× MIC DLP2 or DLP4 at 37°C for 0.5, 1 and 2 h, respectively. After washing twice with PBS, cells were dyed with 50 μg/ml propidium iodide (PI) and analyzed using a BD FACSCalibur flow cytometer (BD, USA). Data were analyzed with Cell Quest Pro software (BD, USA)[5].

Scanning electron microscopy (SEM) observation

Mid-log phase MRSA ATCC43300 suspension (1× 10^8 CFU/ml) were incubated with 2× MIC DLP2 or DLP4 for 2 h at 37°C, centrifuged at 4,000 rpm for 5 min, washed for three times with 0.1 M PBS (pH 7.2) and fixed using 2.5% glutaraldehyde at 4°C for 2 h. After washing three times with PBS, the samples were post-fixed with 1% osmium tetroxide for 2 h, dehydrated by a graded ethanol series (50%~70%~85%~95%~100%), and CO_2 dried. The samples were sputter-coated with platinum and observed using a QUANTA200 SEM (FEI, Philips, Netherlands).

Transmission electron microscopy (TEM) observation

The exponential phase MRSA ATCC43300 (1× 10^8 CFU/ml) cells were treated with 2× MIC DLP2 or DLP4 for 2 h at 37 ºC, centrifuged at 5,500 *g* for 5 min, and washed three times with PBS. The cells were then fixed with 2.5% glutaraldehyde at 4 ºC overnight and dehydrated in a graded series of ethanol. After being air-dried, mounted and sputter-coated with carbon, samples were fixed in 1% buffered osmium tetroxide for 1 h, stained with 1% uranyl acetate, and subsequently dehydrated with a graded ethanol series. Finally, samples were embedded in Spur resin, sectioned, stained with 2% uranyl acetate and lead citrate, and observed using a TEM (JEM-1400, JEDL, Tokyo, Japan).

Interaction of DLP2 and DLP4 with *S. aureus* DNA

Briefly, different concentrations of peptides and genomic DNA were added into 20 μl binding buffer (10 mM Tris-HCl (pH 8.0), 5% glycerol, 1 mM dithiothreitol, 1 mM EDTA, 20 mM KCl and 50 μg/ml bovine serum albumin) according to the ratios of 0, 0.5, 1, 2.5, 5, 10 and 12, respectively (peptide/DNA, w/w). After incubation for 10 min at 37°C, the binding of DLP2/DLP4 and DNA was assessed by electrophoresis on a 1% agarose gel.

Effect of DLP2 and DLP4 on the cell cycle of *S. aureus*

The MRSA ATCC43300 cells (10^8 CFU/ml) were incubated with DLP2 and DLP4 for 0.5~2 h at 37°C.

After centrifugation, the cells were collected, washed with PBS buffer and fixed in cold ethanol (75%) at 4°C overnight. The cells were again collected, resuspended in PBS containing RNase A and incubated for 0.5 h at 37°C. The PI solution (50 μg/ml) was added into cells and stained for 0.5 h in the dark. The DNA contents of cells were examined in a flow cytometer and the cell cycle was analyzed using ModFit LT 4.1 software program.

Effect of DLP2 and DLP4 on the macromolecular synthesis of *S. aureus*

The effects of DLP2 and DLP4 on the incorporation of L-[methyl-^{3}H] thymidine, ^{3}H-uridine, D-[6-^{3}H(N)] glucosamine hydrochloride and ^{3}H-leucine into DNA, RNA, peptidoglycan and protein were investigated in MRSA ATCC43300. Briefly, DLP2 (2× MIC), DLP4 (2× MIC), vancomycin (2× MIC) or ciprofloxacin (8× MIC) were added into the mid-log phase cells (10^{8} CFU/ml) and incubated at 37°C for 15 min. Vancomycin and ciprofloxacin were used as positive controls. The radiolabeled precursor of 3H-thymidine and 3H-glucosamine (40 μCi/ml) were then added to cells and incubated for 20 min at 37°C. Cells were followed by adding cold 25% trichloroacetic acid (TCA) and placing on ice for 30 min. Cells were centrifuged, washed with TCA, and mixed with scintillation fluid. Finally, radioactivity was measured in a MicroBeta 1450 scintillation counter[6].

References:

[1] Zhang, Y. *et al*. High expression of a plectasin-derived peptide NZ2114 in *Pichia pastoris* and its pharmacodynamics, postantibiotic and synergy against *Staphylococcus aureus*. *Appl Microbiol Biotechnol* **98**, 681–694 (2014).

[2] Yu, H. H. *et al*. Antimicrobial activity of berberine alone and in combination with ampicillin or oxacillin against methicillin-resistant *Staphylococcus aureus*. *J Med Food* **8**, 454–461 (2005).

[3] Jung, H. J. *et al*. Fungicidal effect of pleurocidin by membrane-active mechanism and design of

enantiomeric analogue for proteolytic resistance. *Biochim Biophys Acta* **1768**, 1400–1405 (2007).

[4] Mah, T. F. *et al*. A genetic basis for *Pseudomonas aeruginosa* biofilm antibiotic resistance. *Nature* **426**, 306–310 (2003).

[5] Warnes, S. L., Caves, V. & Keevil, C. W. Mechanism of copper surface toxicity in *Escherichia coli* O157:H7 and *Salmonella* involves immediate membrane depolarization followed by slower rate of DNA destruction which differs from that observed for Gram-positive bacteria. *Environ Microbiol* **14**, 1730–1743 (2012).

[6] Xiong, Y. Q., Bayer, A. S. & Yeaman, M. R. Inhibition of intracellular macromolecular synthesis in *Staphylococcus aureus* by thrombin-induced platelet microbicidal proteins. *J Infect Dis* **185**, 348–356 (2002).

[7] Gautier, R., Douguet, D., Antonny, B. & Drin, G. HELIQUEST: a web server to screen sequences with specific alpha-helical properties. *Bioinformatics* **24**, 2101–2102 (2008).

Supplementary 2: Table

Table 1 MIC values of DLP2, DLP4 and antibiotics against Gram-positive bacteria in MHB containing added different concentrations of NaCl

**DLPs and antibiotics**	**Strain**	**MIC (μg/ml)**				
		**+ NaCl (%)**				
		**0**	**0.225**	**0.45**	**0.9**	**1.8**
DLP2	*S. aureus* ATCC25923[a]	0.031	NT	NT	NT	NT
	S. aureus ATCC43300[a]	0.5	0.5	1	0.5	1
	S. aureus ATCC6538[a]	0.5	0.5	1	1	1
	S. aureus CICC546[c]	1	1	1	1	1
	S. suis CVCC606[b]	4	4	2	1	0.5
	L. ivanovii ATCC19119[a]	0.5	1	0.5	0.5	1
DLP4	*S. aureus* ATCC25923[a]	0.062	NT	NT	NT	NT
	S. aureus ATCC43300[a]	1	1	1	1	1
	S. aureus ATCC6538[a]	2	2	2	4	4
	S. aureus CICC546[c]	2	2	2	1	2
	S. suis CVCC606[b]	8	4	4	1	0.5
	L. ivanovii ATCC19119[a]	0.5	0.5	0.5	0.5	1
Vancomycin	*S. aureus* ATCC25923[a]	0.062	NT	NT	NT	NT
	S. aureus ATCC43300[a]	1	1	1	1	1
	S. aureus ATCC6538[a]	NT	NT	NT	NT	NT
	S. aureus CICC546[c]	0.5	1	0.5	1	1
	S. suis CVCC606[b]	0.03	0.015	0.015	0.015	0.015
	L. ivanovii ATCC19119[a]	0.5	0.5	0.5	0.5	1
Ciprofloxacin	*S. aureus* ATCC25923[a]	0.031	NT	NT	NT	NT
	S. aureus ATCC43300[a]	1	2	1	1	2
	S. aureus ATCC6538[a]	NT	NT	NT	NT	NT
	S. aureus CICC546[c]	1	1	1	1	1
	S. suis CVCC606[b]	0.015	0.015	0.015	0.015	0.015
	L. ivanovii ATCC19119[a]	0.25	0.5	0.5	0.5	0.5

Note: NT: not test. [a]American Type Culture Collection (ATCC); [b]China Veterinary Culture Collection Center (CVCC); [c]China Center of Industrial Culture Collection (CICC); [d]National Center Center for Medical Culture Collection (CMCC). Data were representative of three independent experiments.

Supplementary 3: Figures

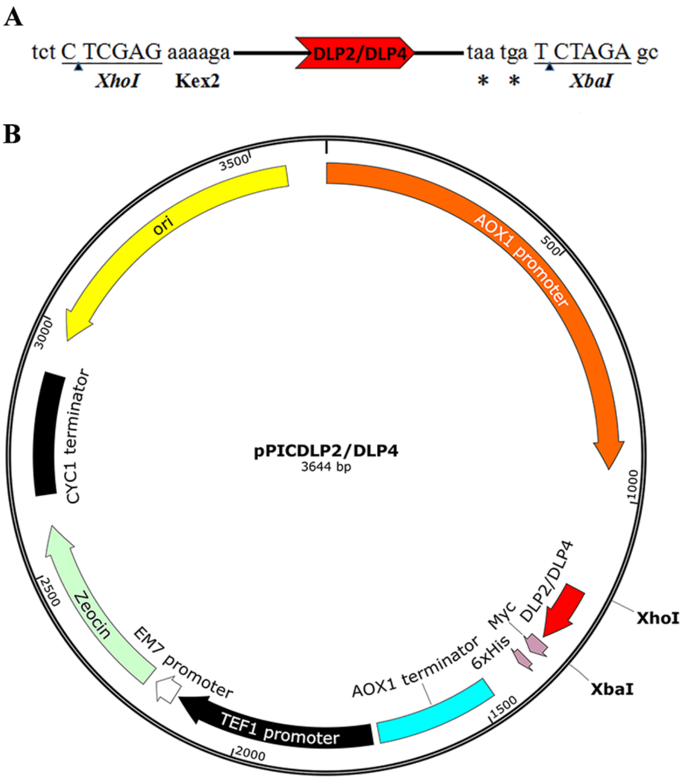

Fig. S1

Figure 1 The construction of the pPICDLP2 and pPICDLP4 plasmids. **(A)** The fragment of peptide genes. Letters underlined are the *Xho*I and *Xba*I restriction endonuclease sites, and the arrows indicate the cleavage sites. The lowercase letters "aaaaga" indicate the Kex2 cleavage site. The red arrow located in the middle indicates the sequence of DLP2 or DLP4. **(B)** Schematic diagram of pPICDLP2 and pPICDLP4 D plasmids using the SnapGene Viewer.

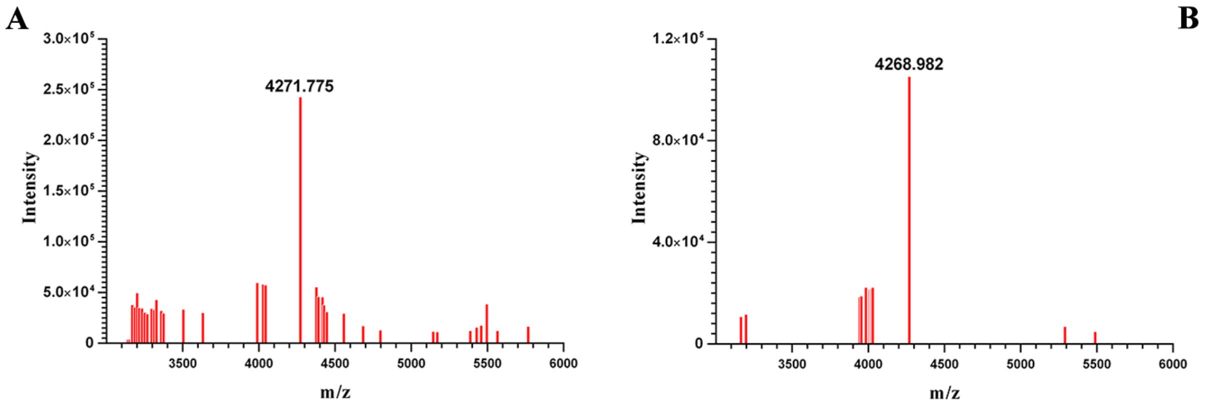

Fig. S2

Figure 2 MALDI-TOF MS analysis of the purified DLP2 and DLP4. **(A)** DLP2. (**B)** DLP4.

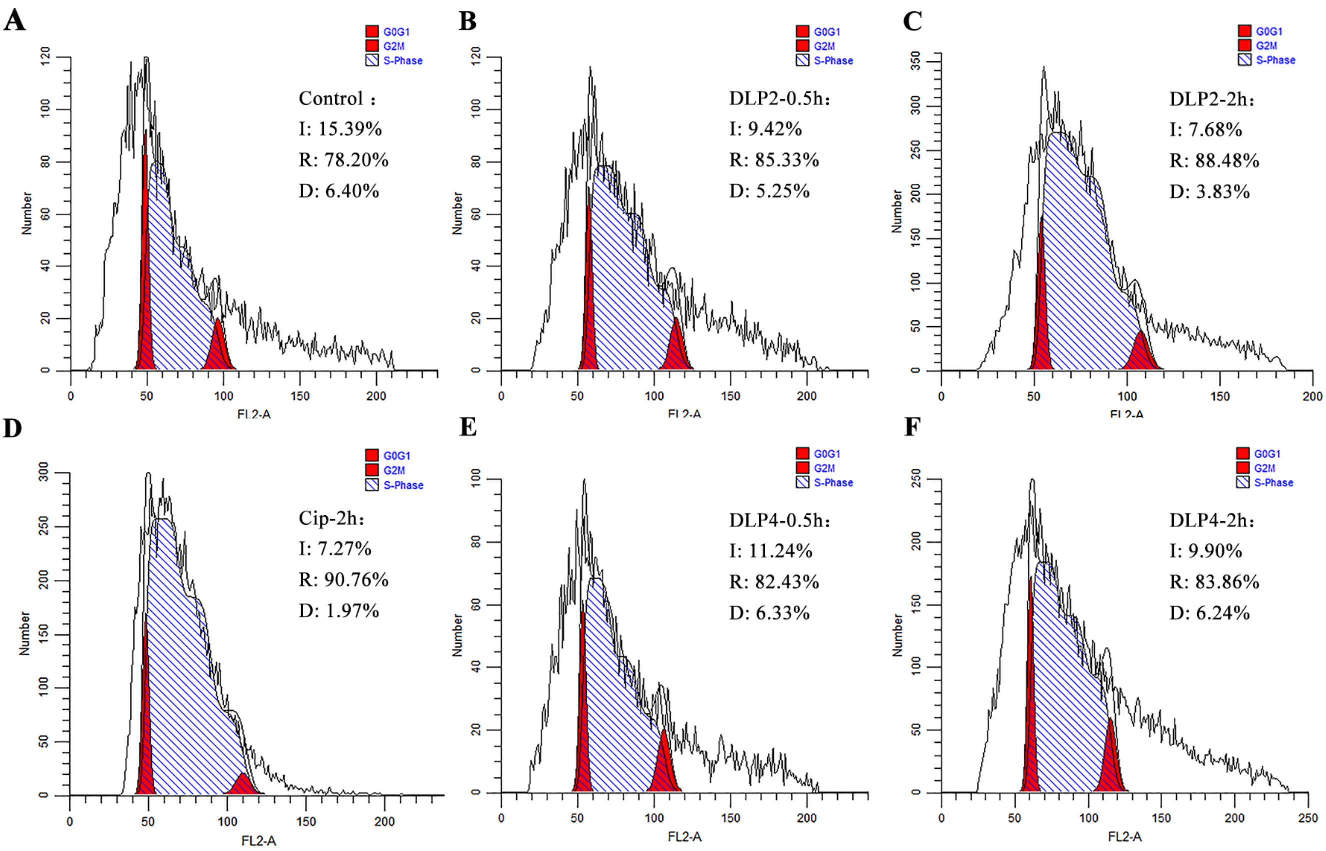

Fig. S3

Figure 3 The effects of DLP2 and DLP4 on the cell cycle of MRSA ATCC43300. *S. aureus* (10^8 CFU/ml) was cultured alone as a control group or cultured in the presence of 2× MIC DLP2, DLP4 or ciprofloxacin for 0.5 h or 2 h, respectively. Ciprofloxacin was used the positive control. The cell cycle distribution was analyzed by a flow cytometry. Cip: ciprofloxacin. **(A)** Untreated cells. **(B, C)** Cells treated with DLP2 for 0.5 h (**B**) and 2 h (**C**). **(D)** Cells treated with ciprofloxacin for 2 h. **(E, F)** Cells treated with DLP4 for 0.5 h (**E**) and 2 h (**F**).

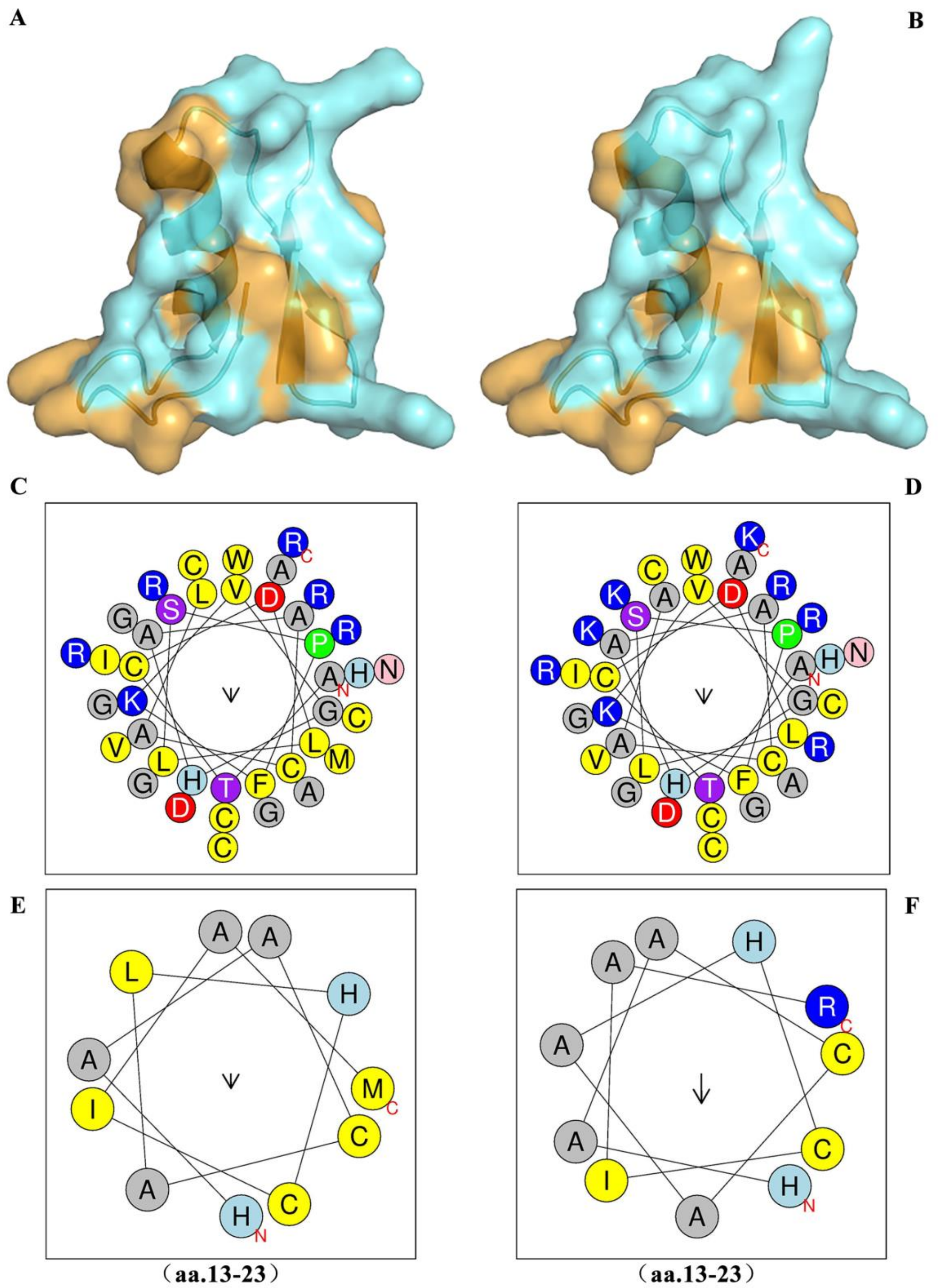

Fig. S4

Figure 4 DLP2 and DLP4 sequence analysis and modeling. **(A, B)** 3D structure of DLP2 (**A**) and DLP4 (**B**) generated using PyMol. **(C-F)** Helical wheel projection of DLP2 (**C, E**) and DLP4 (**D, F**) generated using Heliquest[7]. Alfa spiral full length of DLP2 (**C**) and DLP4 (**D**) and partial of DLP2

(hydrophobicity of 0.846) and DLP4 (hydrophobicity of 0.516) were predicted by SWISS-MODEL Workspace (http://swissmodel.expasy.org/workspace/). The arrows indicate the helical moment. Gray and yellow: the nonpolar residues; cyan: uncharged and polar residues; blue: the charged residues.

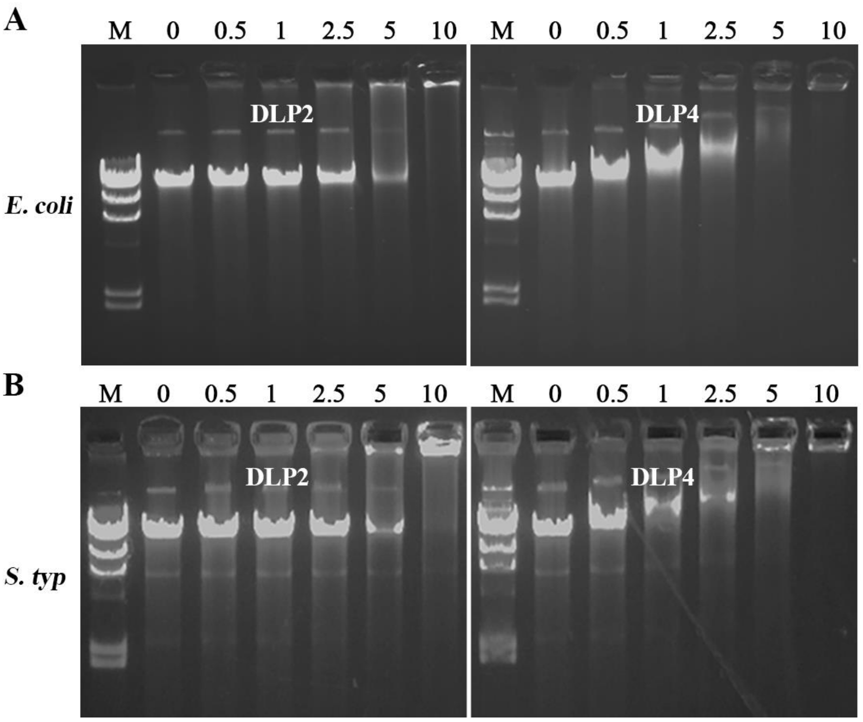

Fig. S5

Figure 5-1 Gel retardation analysis of binding of DLP2 and DLP4 to bacterial genomic DNA. Marker, λ *hind*III. Lanes 2 to 7, the mass ratios of DLP2 and genomic DNA from *E. coli* CVCC1515 (**A**) were 0, 0.5, 1, 2.5, 5, and 10, respectively. Lanes 9 to 14, the mass ratios of DLP4 and genomic DNA from *S. typhimurium* ATCC14028 (**B**) were 0, 0.5, 1, 2.5, 5, and 10, respectively.

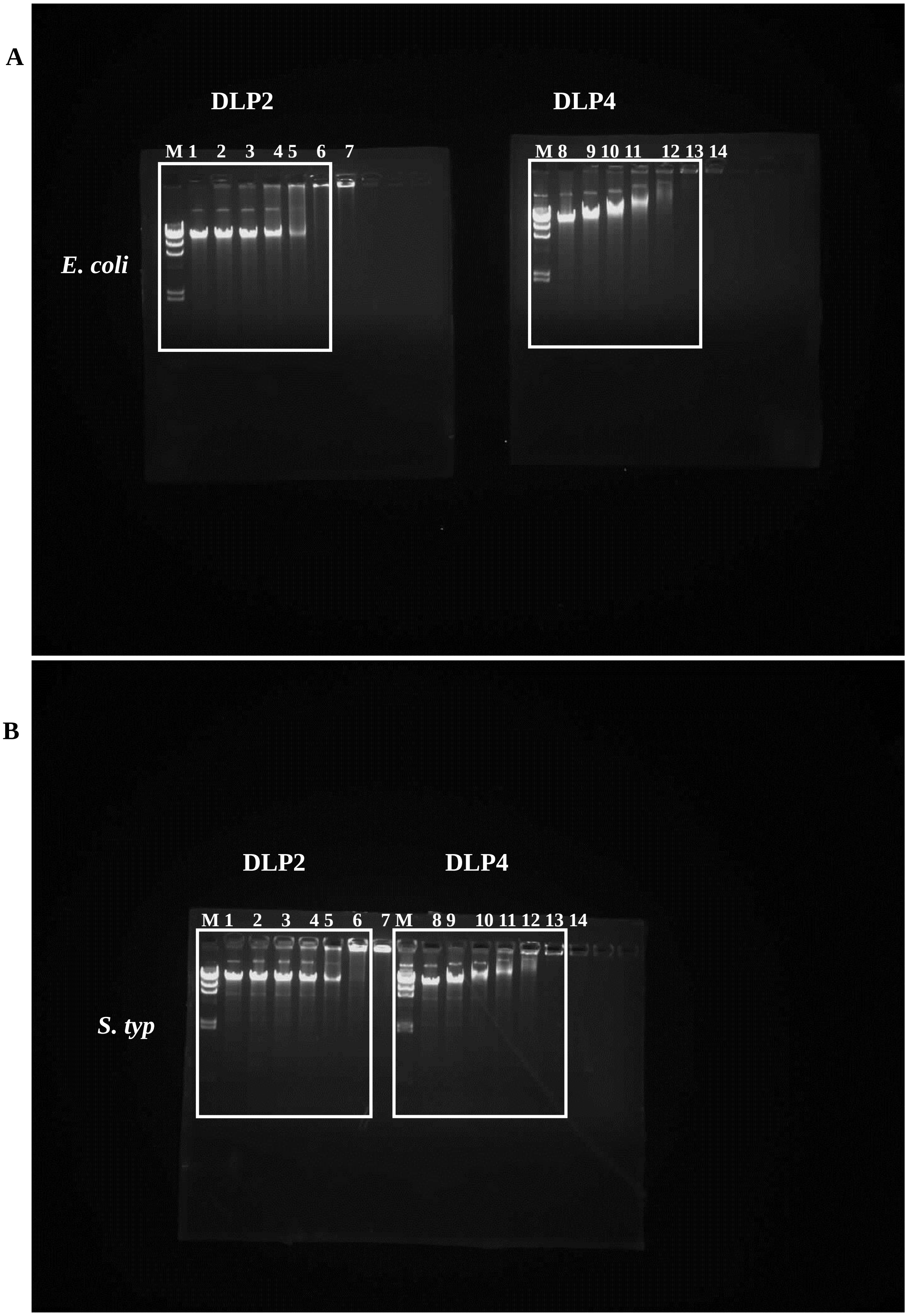

Fig. S5

Figure 5-2 Full-length gel retardation analysis of binding of DLP2 and DLP4 to bacterial genomic

DNA. M, λ *hind*III marker. Lanes 1 to 7, the mass ratios of DLP2 and genomic DNA from *E. coli* CVCC1515 (**A**) were 0, 0.5, 1, 2.5, 5, 10, and 12, respectively. Lanes 8 to 14, the mass ratios of DLP4 and genomic DNA from *S. typhimurium* ATCC14028 (**B**) were 0, 0.5, 1, 2.5, 5, 10, and 12, respectively.

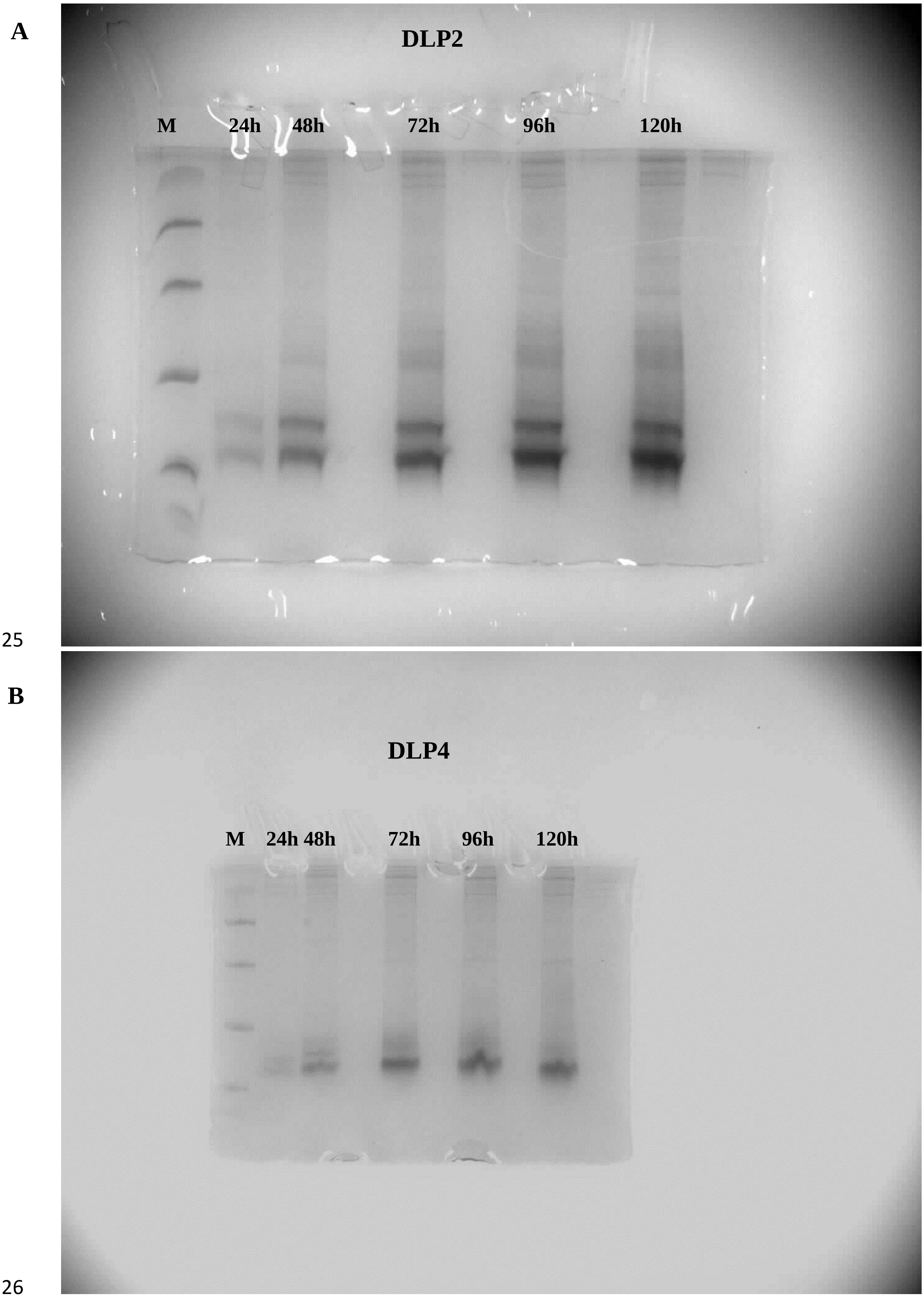

Fig. S6

Figure 6 Full-length gel analysis of the fermentation supernatants at different induced time. Lanes M:

protein molecular weight marker (5 μl); others lanes: fermentation supernatants (10 μl) of DLP2 (**A**) and DLP4 (**B**) were taken at 0, 24, 48, 72, 96 h, respectively.

A

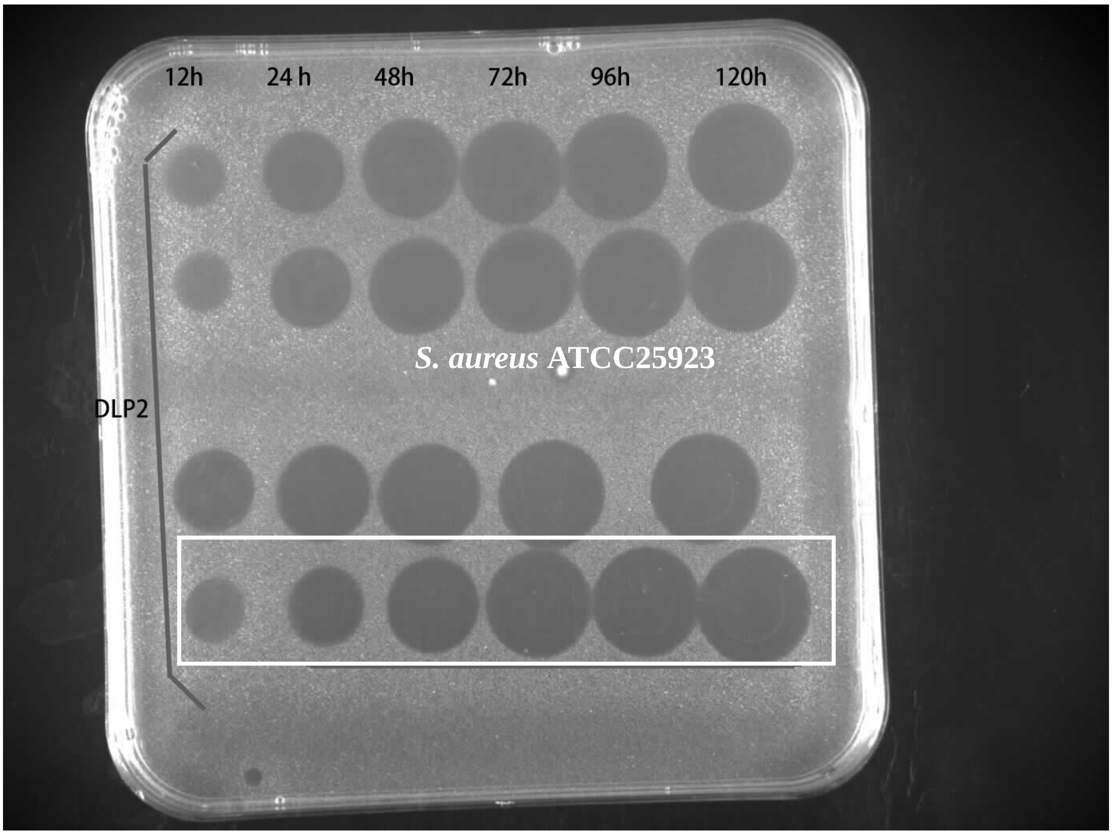
12h
24 h
48h
72h
96h
120h
S. aureus ATCC25923
DLP2

B

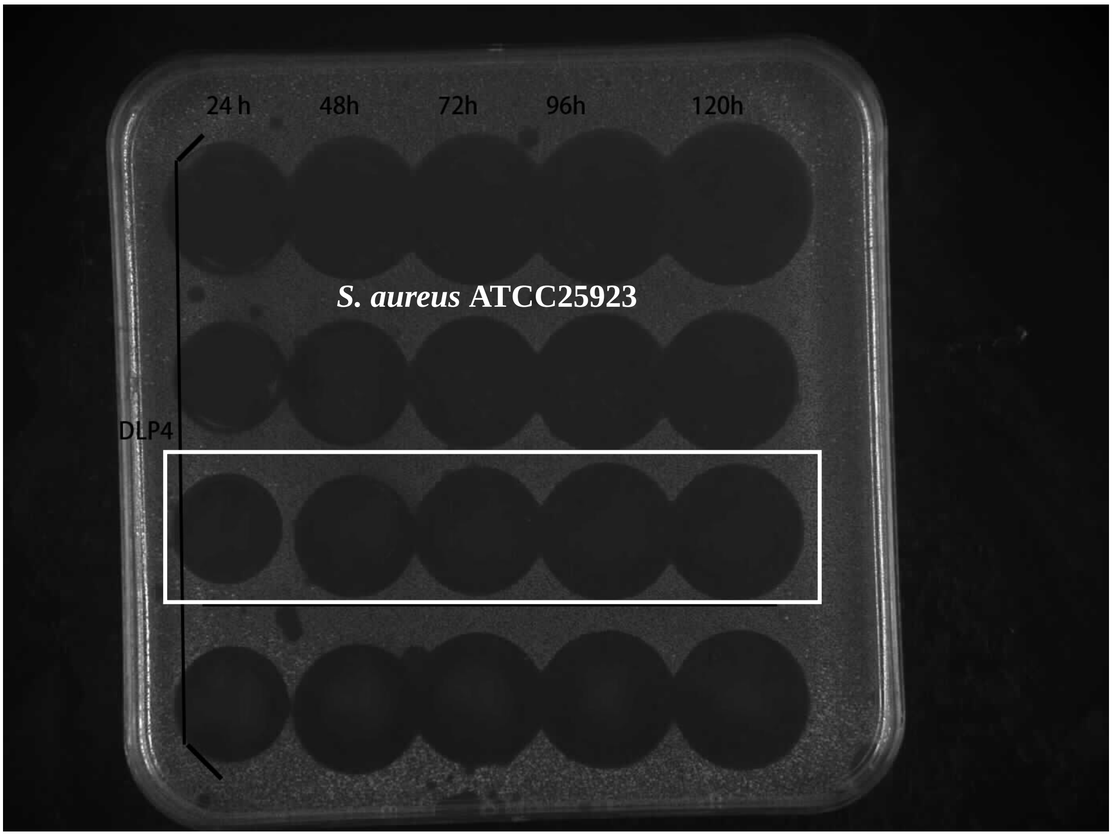
24 h
48h
72h
96h
120h
S. aureus ATCC25923
DLP4

C

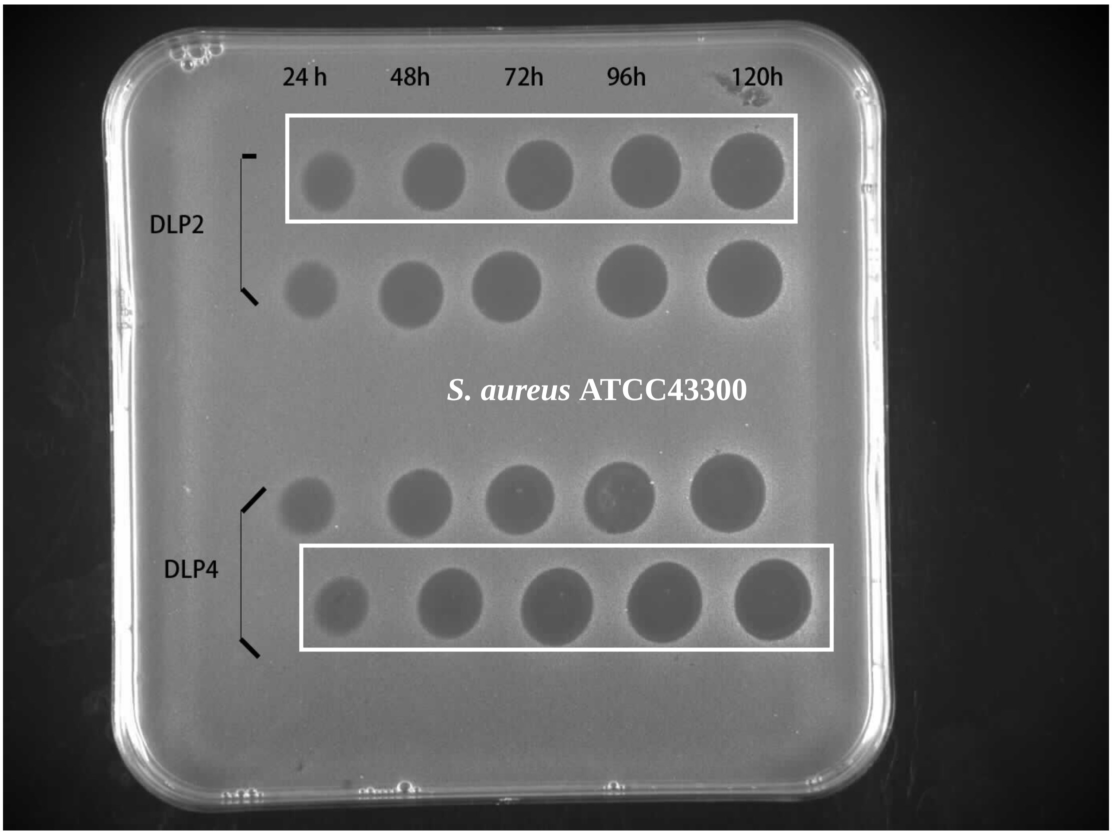

Fig. S7

Figure 7 The full-length inhibition zones of fermentation supernatant against *S. aureus* ATCC25923 (**A, B**) and MRSA ATCC43300 (**C**). Fermentation supernatants of DLP2 (12 μl) (**A, C**) and DLP4 (20 μl) (**B, C**) were taken at 0, 24, 48, 72, 96, and 120 h, respectively.

A

B

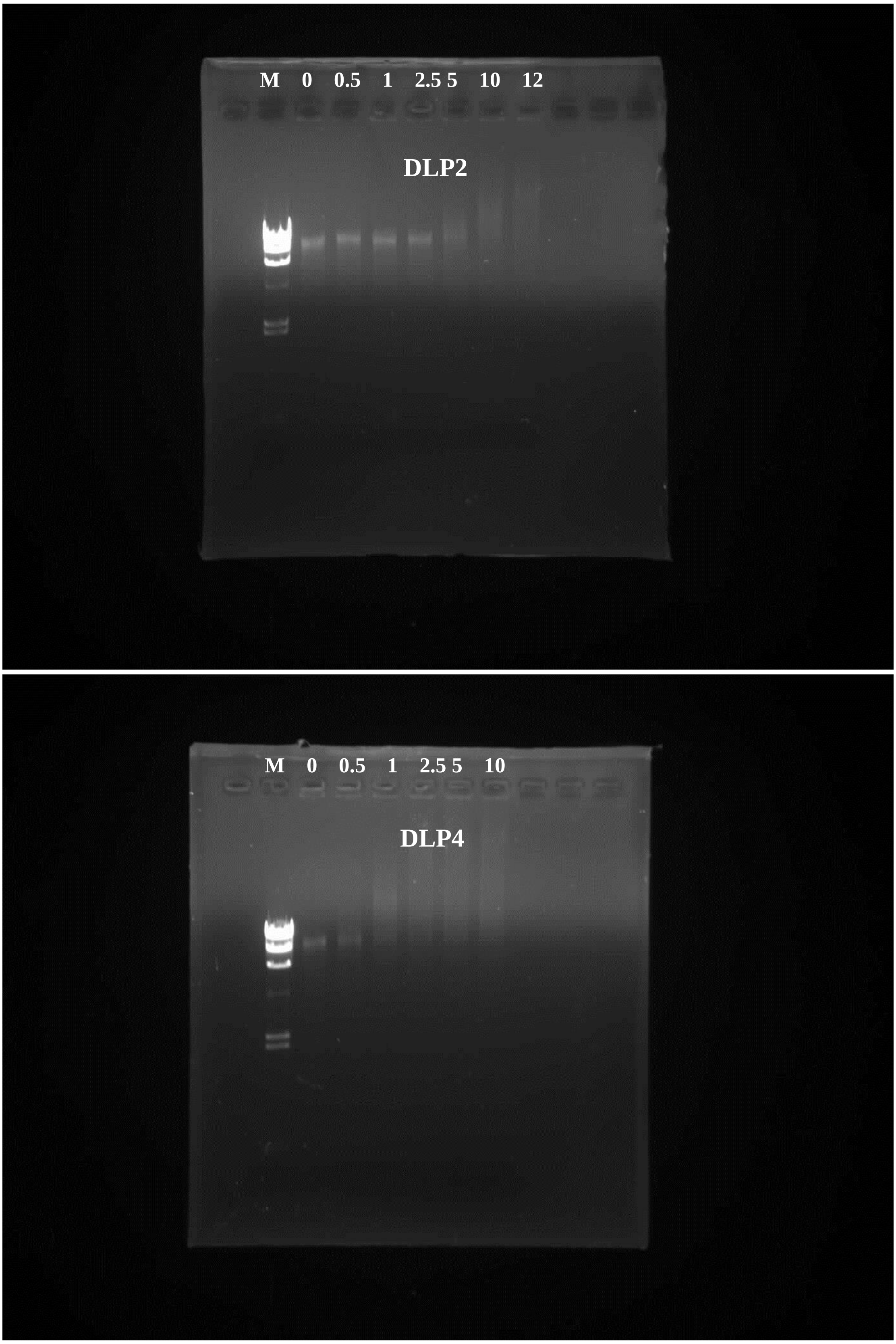

Fig. S8

Figure 8 Full-length gel retardation analysis of binding of DLP2 (**A**) and DLP4 (**B**) to DNA. Marker, λ

*hind*III. Lanes 2 to 8, the mass ratios of DLP2 (**A**) and genomic DNA from MRSA ATCC43300 were 0, 0.5, 1, 2.5, 5, 10, and 12, respectively. Lanes 9 to 14, the mass ratios of DLP4 (**B**) and genomic DNA from *S. aureus* were 0, 0.5, 1, 2.5, 5, and 10, respectively.
